# *In vitro* modeling of polyclonal infection dynamics within the human airways by *Haemophilus influenzae* differential fluorescent labeling

**DOI:** 10.1128/spectrum.00993-23

**Published:** 2023-10-05

**Authors:** Beatriz Rapún-Araiz, Ioritz Sorzabal-Bellido, Javier Asensio-López, María Lázaro-Díez, Mikel Ariz, Carlos Sobejano de la Merced, Begoña Euba, Ariadna Fernández-Calvet, Ivan Cortés-Domínguez, Saioa Burgui, Alejandro Toledo-Arana, Carlos Ortiz-de-Solórzano, Junkal Garmendia

**Affiliations:** 1 Instituto de Agrobiotecnología, Consejo Superior de Investigaciones Científicas (IdAB-CSIC)-Gobierno de Navarra, Mutilva, Spain; 2 Conexión Nanomedicina CSIC (NanomedCSIC), Mutilva, Spain; 3 Laboratorio de Sistemas Microfisiológicos y Biología Cuantitativa, Programa de Ingeniería Biomédica, Centro de Investigación Médica Aplicada (CIMA), Pamplona, Spain; 4 Asociación de la Industria Navarra (AIN)-Gobierno de Navarra, Cordovilla, Spain; 5 Centro de Investigación Biomédica en Red de Enfermedades Oncológicas (CIBERONC), Madrid, Spain; 6 Instituto de Investigación Sanitaria de Navarra (IdiSNA), Pamplona, Spain; 7 Centro de Investigación Biomédica en Red de Enfermedades Respiratorias (CIBERES), Madrid, Spain; Griffith University - Gold Coast Campus, Southport, Gold Coast, Australia

**Keywords:** *Haemophilus influenzae*, plasmid toolbox, fluorescent labeling, mixed biofilms, selective antibiotic efficacy, mixed epithelial infection, polyclonality assessment

## Abstract

Standardized clinical procedures for antibiotic administration rely on pathogen identification and antibiotic susceptibility testing, often performed on single-colony bacterial isolates. For respiratory pathogens, this could be questionable, as chronic patients may be persistently colonized by multiple clones or lineages from the same bacterial pathogen species. Indeed, multiple strains of nontypeable *Haemophilus influenzae*, with different antibiotic susceptibility profiles, can be co-isolated from cystic fibrosis and chronic obstructive pulmonary disease sputum specimens. Despite this clinical evidence, we lack information about the dynamics of *H. influenzae* polyclonal infections, which limits the optimization of therapeutics. Here, we present the engineering and validation of a plasmid toolkit (pTBH, toolbox for *Haemophilus*), with standardized modules consisting of six reporter genes for fluorescent or bioluminescent labeling of *H. influenzae*. This plasmid set was independently introduced in a panel of genomically and phenotypically different *H. influenzae* strains, and two of them were used as a proof of principle to analyze mixed biofilm growth architecture and antibiotic efficacy, and to visualize the dynamics of alveolar epithelial co-infection. The mixed biofilms showed a bilayer architecture, and antibiotic efficacy correlated with the antibiotic susceptibility of the respective single-species strains. Furthermore, differential kinetics of bacterial intracellular location within subcellular acidic compartments were quantified upon co-infection of cultured airway epithelial cells. Overall, we present a panel of novel plasmid tools and quantitative image analysis methods with the potential to be used in a whole range of bacterial host species, assay types, and/or conditions and generate meaningful information for clinically relevant settings.

**IMPORTANCE:**

Genomic diversity of nontypeable *H. influenzae* strains confers phenotypic heterogeneity. Multiple strains of *H. influenzae* can be simultaneously isolated from clinical specimens, but we lack detailed information about polyclonal infection dynamics by this pathogen. A long-term barrier to our understanding of this host-pathogen interplay is the lack of genetic tools for strain engineering and differential labeling. Here, we present a novel plasmid toolkit named pTBH (toolbox for *Haemophilus*), with standardized modules for fluorescent or bioluminescent labeling, adapted to *H. influenzae* requirements but designed to be versatile so it can be utilized in other bacterial species. We present detailed experimental and quantitative image analysis methods, together with proof-of-principle examples, and show the ample possibilities of 3D microscopy, combined with quantitative image analysis, to model *H. influenzae* polyclonal infection lifestyles and unravel the co-habitation and co-infection dynamics of this respiratory pathogen.

## INTRODUCTION

Sampling of bacterial pathogens and selecting appropriate antibiotic regimes for treating bacterial infectious diseases often rely on antimicrobial susceptibility profiling of single colonies isolated from cultured clinical samples (1). This individual colony sampling may be of limited clinical value as there is evidence of polyclonality, i.e., coexisting lineages of the same species. This is the case for *Stenotrophomonas maltophilia* and *Staphylococcus aureus* in chronically infected cystic fibrosis (CF) (2, 3) or for *Haemophilus influenzae* in chronically infected CF or chronic obstructive pulmonary disease (COPD) patients (4 – 7). Multiple coexisting strains may have distinct phenotypes as shown for *S. maltophilia* (2) and often different antibiotic susceptibility profiles, as reported for *S. aureus* and *H. influenzae* (3, 4), with important implications for antibiotic decision making. Despite existing clinical evidence, our understanding of polyclonal infection dynamics and consequences for the infected patients is limited and, therefore, a barrier for optimized therapeutics.

*H. influenzae* is a human-adapted Gram-negative bacterial pathobiont that causes infections in susceptible hosts. The high efficacy of the *H. influenzae* type b (Hib) vaccine has almost completely eradicated this serotype in countries with established child-immunization programs, while nontypeable strains (NTHi) have emerged, causing otitis, conjunctivitis, sinusitis, and lower respiratory infections in children; exacerbations of COPD and CF in adults; and invasive disease in neonates, immunocompromised adults, and the elderly (8 – 11). NTHi is an intracellular facultative pathogen, capable of both forming biofilms and invading airway epithelial cells, a combination of pathoadaptive traits likely contributing to persistence (12 – 16). Multiple strains of *H. influenzae* have been simultaneously isolated from longitudinally collected CF and COPD sputum specimens and from nasopharyngeal swabs from infants at risk of otitis media (4 – 7, 17). Polyclonality has also been found in throat cultures sampled from healthy children (18). Moreover, differences in the susceptibility of different strains present within the same COPD sputum have been reported for a variety of antibiotics including ampicillin (4), which may exacerbate the clinical problem as ampicillin-resistant *H. influenzae* is included in the WHO global priority list of bacteria for which new antibacterials are urgently needed (19). In this context, the lack of detailed information about polyclonal infection dynamics by this pathogen limits our understanding of NTHi infection. This issue needs to be addressed to allow the development of efficient counteracting measures.

Polyclonal infections can be assessed at the single-cell level using bacterial strains engineered for differential labeling, but appropriate genetic tools amenable for *H. influenzae* are currently limited. Indeed, the available tools are mostly designed to generate mutant strains or to allow stable chromosomal insertion for mutant complementation by homologous recombination (20, 21). However, plasmid tools designed in a modular manner to ensure enough versatility, suitable to be used in *H. influenzae*, are not available. In this regard, modular plasmid architectures are useful to build, share and compare biological parts and functional vectors, and outperform traditional toolkits as standardization of parts and components streamline assembly (22 – 24). Here, we present a novel genetic toolkit for engineering *H. influenzae*, with functional components (cargo module, two selectable markers, and a replication origin), assembled in modular combinations to create standardized and versatile plasmids. By using this structure, six reporter genes for fluorescent or bioluminescent labeling, expressed from a constitutive promoter, were independently assembled. This plasmid set was introduced in a panel of different *H. influenzae* strains, and two of them were used to study mixed infection by phenotypically divergent strains. This new toolkit, along with quantitative image analysis methods, can provide novel insights on *H. influenzae* polyclonal infection dynamics and antibiotic efficacy.

## MATERIALS AND METHODS

### Bacterial strains and growth conditions

Strains used in this study are listed in Table S1. *H. influenzae* strains were grown at 37°C, 5% CO_2_ on PolyVitex agar (PVX, bioMérieux, 43101), or on *Haemophilus* Test Medium agar (HTM, Oxoid, CM0898) supplemented with 10 µg/mL hemin and 10 µg/mL nicotinamide adenine dinucleotide, referred to as sHTM agar. NTHi liquid cultures were grown at 37°C, 5% CO_2_ in brain-heart infusion (BHI, Oxoid, CM1135) supplemented as indicated above, referred to as sBHI. Erythromycin 11 µg/mL (Erm_11_) was used when necessary. *Escherichia coli* was grown on Luria-Bertani (LB) or LB agar at 37°C, with ampicillin 100 µg/mL (Amp_100_), spectinomycin 50 µg/mL (Spec_50_), or kanamycin 50 µg/mL (Km_50_), when necessary. *H. influenzae* strains were grown on PVX agar or on sHTM agar with the respective antibiotic, for 16 h. When needed, bacterial biomass bioluminescence was recorded using a ChemiDoc imaging system (Bio-Rad). Planktonic growth was monitored as follows: two to five colonies were inoculated in 10-mL sBHI (containing Erm_11_ when needed) and incubated for 12 h with shaking (100 rpm). Cultures were then diluted to OD_600_ of 0.1 in sBHI (with Erm_11_ when needed), and 200-µL aliquots were transferred to individual wells in polystyrene 96-well flat bottom plates (Sarstedt, 82.1581.001). Plates were incubated at 37°C for 16 h in a SynergyH1 (BioteK) microplate reader; OD_600_ was determined every 30 min. Experiments were performed in triplicate on at least three independent occasions (*n* ≥ 3). Alternatively, bacterial morphology and fluorescence upon planktonic growth were monitored as follows: two to five colonies were inoculated in 10-mL sBHI containing Erm_11_ and incubated for 12 h with shaking (100 rpm). Cultures were diluted to OD_600_ of 0.07 in 20-mL of fresh sBHI containing Erm_11_ and incubated at 37°C, 5% CO_2_ in agitation up to OD_600_ of 0.3. One milliliter per sample was then collected and centrifuged (4,000 rcf, 15 min, 4°C). Pellets were washed twice with phosphate buffered saline (PBS), resuspended in 250-µL 3% paraformaldehyde, and incubated at room temperature (RT) for 20 min. Fixed bacterial suspensions were washed twice with PBS, and pellets were resuspended into 200-µL PBS. Three to five microliters per sample were spotted onto glass coverslips slides, air dried, and mounted with EverBrite Hardset Mounting with DAPI (Biotium, 23004). Representative images were captured using a Zeiss LSM 880 AxioObserver inverted confocal microscope equipped with a 63× Plan-Aprochomat 1.4 NA oil immersion objective. For completion, upon cultures reaching OD_600_ of 0.3, 2 mL per sample was collected and centrifuged (14,000 rcf, 5 min). Pellets were washed with PBS and resuspended in 1-mL PBS, and 100-µL aliquots were transferred to individual wells in 96-well plates (Nunc Optical Bottom plates with opaque polystyrene, Thermo Fisher Scientific, 165305) for fluorescence quantification in a SynergyH1 microplate reader. The fluorescence signal of each fluorescent protein was quantified using a monochromator-based setting with specific excitation and emission wavelengths: green fluorescence protein (GFP) at 485/515 nm, mCherry and mPlum at 587/645 nm, mOrange at 520/570 nm, and cCFP at 435/485 nm. When necessary, bioluminescence was quantified by using a luminescence filter cube (BioteK, 8040553) included in the SynergyH1 microplate reader, maintaining default settings. Assays were performed in triplicate on two independent occasions (*n* = 2).

### Antibiotic minimal inhibitory concentration (MIC)

Ampicillin, cefuroxime, cefepime, ceftriaxone, imipenem, meropenem, chloramphenicol, tetracycline, azithromycin, rifampicin, nalidixic acid, and levofloxacin susceptibility of strains *H. influenzae* RdKW20 and R2866 was determined by using the microdilution broth method, following EUCAST guidelines (1). Ampicillin susceptibility of the RdKW20-pTBH03 and R2866-pTBH05 plasmid transformed strains was determined by microdilution broth method, following EUCAST guidelines with some modifications. Briefly, ampicillin was used by freshly preparing a 100 mg/mL stock solution in distilled H_2_O (dH_2_O), diluted to 512 µg/mL in sBHI. Next, 200 µL of ampicillin 512 µg/mL was added to individual wells in row A in 96-well plates. One-hundred-microliter aliquots of sBHI were transferred to individual wells in the rest of the plate. Next, 100-µL aliquots were serially transferred from wells in row A to wells in row B and up to row G, where 100 µL was discarded. A suspension of bacteria grown on HTM agar containing Erm_11_ was generated with fresh sBHI, adjusted to 0.5 MacFarland (OD_600_ = 0.063) and diluted 1:100. sBHI containing Erm 22 µg/mL was used for strains carrying toolbox for *Haemophilus* (pTBH) plasmids. Next, 100-µL bacterial aliquots were transferred to each well (Erm well working concentration was 11 µg/mL for strains carrying pTBH plasmids). Plates were incubated for 24 h at 37°C, 5% CO_2_, without shaking, and MIC was determined. Bacterial growth controls (without antibiotics) were included in each case (row H). Assays were carried out in triplicate on three independent occasions (*n* = 3).

### pTBH plasmid series generation and stability

All plasmids were generated by standard cloning techniques. Details are available in the Results section and in Table 1. Phusion polymerase and primers listed in Table S2 were used, the resulting DNA fragments were purified with a PCR and gel purification kit (Macherey-Nagel, 22740609.250) following the manufacturer’s instructions, and appropriate FastDigest restriction enzymes and “Rapid DNA ligation kit” (Thermo Fisher Scientific, K1423) were used. *E. coli* TOP10 was used as an intermediate host; plasmids were extracted and purified (Qiagen midiprep kit, 12143) and transformed into *H. influenzae* electrocompetent cells (25). Transformants were selected on sHTM agar containing Erm_11_ and confirmed by PCR.

**TABLE 1:** Plasmids used in this study

Plasmids	Description	Source
pJET1.2/blunt	Cloning vector, Amp^R^	Fisher Scientific
pRSM2211	Plasmid containing the *gfpmut3* gene, under the promoter of the *ompP2* gene (Km^R^)	(25)
pCN47	*E. coli-S. aureus* shuttle vector for cloning. Amp^R^, Erm^R^	(26)
pACYC177	Cloning vector, Amp^R^, Cm^R^	New England Biolabs
pHRG	pCN47 plasmid carrying Phyper constitutive promoter, *icaR* RBS, and the *gfpmut2* gene	(27)
pSEVA236	Cloning vector from the pSEVA collection, containing the *luxCDABE* operon (5,798 bp), a Km^R^ gene, and pBBR1 origin of replication	https://seva-plasmids.com
pHRR	pCN47 plasmid carrying the Phyper constitutive promoter, *icaR* RBS and the *mcherry* reporter gene	(28)
pCN47-Phyper-cCFP	pCN47 plasmid expressing the *ccfp* gene, under the Phyper promoter	Toledo-Arana
pGT-mPlum	pGEM-T derivative carrying promoter-less ORF *mplum*	(29)
pGT-mOrange2	pGEM-T derivative carrying promoter-less ORF *morange*2	(29)
pTBH01	pACYC177 derivative containing an Erm^R^ gene and an MCS (Amp^R^ , Erm^R^)	(15)
pTBH02	pTBH01 derivative, expressing *gfpmut*2 under the Phyper promoter with *icaR* RBS. A fragment of 792 bp was digested with *Sph*I and *Asc*I from pHRG and religated into pTBH01(Amp^R^, Erm^R^).	This study
pTBH03-*Pr_hmw_*	pTBH-01 derivative containing a *Pr_hmw_::gfp* transcriptional fusion	(15)
pTBH03	pTBH03-*Pr_hmw_* derivative, expressing *gfpmut*2 under the Phyper promoter, with Gram-negative RBS. Phyper (48 bp) was digested with *Sph*I and *EcoR*I from pHRG and cloned into pTBH03-*Pr_hmw_*.	This study
pTBH03-Prom-less	pTBH01 derivative, containing the *gfpmut*2 gene, without promoter. A fragment of 750 bp was digested with *EcoR*I and *Asc*I from pTBH03 and ligated into pTBH01 (Amp^R^, Erm^R^).	This study
pTBH04	pTBH03 derivative, expressing *mcherry* under the Phyper promoter with Gram-negative RBS. pHRR was used as template for PCR with primers #2158 and #2159. The PCR fragment (720 bp) was digested with *Spe*I and *Asc*I and ligated into pTBH3 (Amp^R^, Erm^R^).	This study
pTBH04-Prom-less	pTBH01 derivative, containing the *mcherry* gene without promoter. A 741-bp fragment was digested with *EcoR*I and *Asc*I from pTBH04 and ligated into pTBH01 (Amp^R^, Erm^R^).	This study
pTBH05	pTBH03 derivative expressing the *mplum* gene under the Phyper promoter with Gram-negative RBS. pGT-mPlum was used as template for PCR with primers #2194 and #2195. The PCR fragment (693 bp) was digested with *Spe*I and *Asc*I and ligated into pTBH03 (Amp^R^, Erm^R^).	This study
pTBH06	pTBH03 derivative expressing the *morange*2 gene under the Phyper constitutive with Gram-negative RBS. pGT-mOrange2 was used as template for PCR with primers #2159 and #2196. The PCR fragment (720 bp) was digested with *Spe*I and *Asc*I and ligated into pTBH03 (Amp^R^, Erm^R^).	This study
pTBH07	pTBH03 derivative expressing the *ccfp* gene under the Phyper promoter with Gram negative RBS. pCN47-Phyper-cCFP was used as template for PCR with primers #2160 and #2161. The PCR fragment (729 bp) was digested with *Spe*I and *Asc*I and ligated into pTBH03 (Amp^R^, Erm^R^).	This study
pTBH08	pTBH03 derivative expressing the *luxCDABE* operon under the Phyper promoter. pSEVA236 was used as template for PCR with primers #2178 and #2179. The PCR fragment (5,830 bp) was digested with *EcoR*I and *Asc*I and ligated into pTBH03 (Amp^R^, Erm^R^).	This study

The curing rate of pTBH01 plasmid in *H. influenzae* RdKW20 and R2866 transformants was determined as previously described (30). Briefly, 4 to 5 colonies of bacteria harboring pTBH01 grown on sHTM agar containing Erm_11_ were inoculated in 10-mL sBHI and grown at 37°C in 5% CO_2_ for 12 h. This culture was serially passed eight times by serial 1:100 dilution in 10-mL sBHI and growth for 12 h. In each subculture step, bacteria were plated on sHTM agar, and the proportion of resistant colonies harboring pTBH01 was deduced by replica plating of at least 100 colonies on sHTM agar and sHTM agar plates containing Erm_11_. The rate of plasmid curing was calculated as the percentage of erythromycin-resistant colonies at each cycle per total number of colonies replicated at each cycle from three independent experiments.

### Biofilm growth determined by crystal violet staining

*H. influenzae* strains were grown on PVX agar or on sHTM agar with Erm_11_ for 16 h and then overnight cultured in sBHI containing Erm_11_ when needed. Overnight grown cultures were diluted to OD_600_ of 0.05 in fresh sBHI (containing Erm_11_ for strains carrying pTBH plasmids), transferring 150 µL per well of diluted cultures to polystyrene 96-well flat bottom plates. Plates were incubated at 37°C with 5% CO_2_ for 24 h without shaking. Bulk bacterial biomass was assessed by measuring OD_600_ on a SynergyH1 (Biotek) microplate reader. The liquid portion in each well was then discarded, and plates were washed three times by gently pipetting 150-µL dH_2_O/well and allowed to air dry. Next, 150 µL/well 0.5% crystal violet (Merck, V5265) was added, and plates were incubated for 20 min at RT on gentle agitation, followed by plate washing as previously described. Finally, 150 µL/well 95% ethanol was added, plates were incubated for 20 min at RT on gentle agitation, and OD_570_ was determined as a measure of biofilm biomass. The OD_570_/OD_600_ ratio for each strain from at least three independent assays (*n* ≥ 3) was calculated to correlate biofilm biomass to overall growth.

### Biofilm growth determined by confocal laser scanning microscopy (CLSM)

*H. influenzae* biofilms were imaged by CLSM in four different experimental settings. (i) In Live-Dead staining, *H. influenzae* strains were grown on PVX agar or on sHTM agar with Erm_11_, for 16 h, and overnight cultured in sBHI containing Erm_11_ when needed. Cultures were then diluted to OD_600_ of 0.05 in fresh sBHI (containing Erm_11_ for strains carrying pTBH plasmids), transferring 250 µL of diluted cultures to each well of an eight-well chambered coverglass-bottomed device for confocal image analysis (Ibidi GmbH). Devices were incubated at 37°C with 5% CO_2_ for 24 h without shaking. The medium was then exchanged by 200-µL saline solution (NaCl 0.85%) with 0.3-µL SYTO9 for staining live bacteria and 0.3-µL propidium iodide (PI) for staining dead bacteria (*Bac*Light, Invitrogen) and incubated for 20 min at RT prior to CLSM analysis. (ii) In SYTO9 staining, *H. influenzae* cultures were prepared, and biofilms were grown on chambered coverglass-bottomed devices as described in (i). After biofilm growth, medium was exchanged by 200-µL NaCl 0.85% with 0.3-µL SYTO9 for staining and incubated for 20 min at RT prior to CLSM analysis. (iii) In biofilm formation by fluorescently labeled bacteria, *H. influenzae* cultures were prepared, and biofilms were grown on chambered coverglass-bottomed devices as described in (i). For imaging purposes, sBHI was replaced by PBS, and fluorescent bacteria were observed by CLSM. (iv) In biofilm formation under the effect of antibiotics, bacteria freshly grown on HTM agar containing Erm_11_ were used to generate suspensions in sBHI containing Erm_11_, adjusted to 0.5 MacFarland (OD_600_ = 0.063), and diluted 1:200. Next, 125 µL of each diluted culture were transferred to each well of eight-well chambered coverglass-bottomed devices. Ampicillin was added (working concentrations indicated in the Results section), and chambers were incubated at 37°C with 5% CO_2_ for 24 h without shaking. sBHI was then exchanged by PBS, and fluorescent bacterial biofilms were analyzed by CLSM.

CLSM image acquisition was performed using a Zeiss LSM 880 AxioObserver inverted confocal microscope equipped with an LD LCI Plan-Apochromat 40 × 1.2 NA W objective. Five Z-stack images (optimal inter-slice separation of 0.48 microns) were acquired for each sample, each image being captured at the center of a well. In the Live-Dead and SYTO9-only experiments, 488-nm and 561-nm excitation lasers were used for SYTO9 and PI, respectively. In single strain and mixed biofilms, GFP-expressing bacteria were imaged at 488-nm excitation wavelength, while CFP-expressing bacteria were visualized at 760 nm (two-photon excitation). Bacteria expressing mOrange, mCherry, and mPlum were excited at 543 nm. Each assay was replicated in two separate experiments, which included duplicate wells for each strain. CLSM stacks were processed with fully automated in-house software.

Specifically, three plugins were developed for Fiji/ImageJ, an open-source Java-based image processing software (31). (i) In analysis of single-strain biofilms, image stacks were first enhanced by means of a median filter and unsharp masking. Bacteria were then segmented using an adaptative percentile threshold for each slice within the Z-stack. The adaptative percentile threshold method segments based on the 5% top tail of the background histogram. The resulting mask was further denoised and refined. Planktonic bacteria were removed from the mask using a logic and operation between consecutive slices. Biofilm growth, i.e., the bacterial capacity for generating a biofilm, was calculated as the relative area (relative to the area of the field of view) occupied by the fluorescent signal within the densest slice of each 3D biofilm image stack, averaged for all the images taken for a given sample (32). Both RdKW20-pTBH06 and R2866-pTBH06 mOrange images stacks were contrast enhanced (saturation of 0.35% of the stack pixels) due to the low signal presented during image acquisition. (ii) In analysis of Live-Dead experiments, live and dead fluorescent signals were separately processed with the same image processing pipeline described above; the resulting masks were processed as described in the previous section to measure the live and dead biofilm fraction as the relative bacterial area within the densest slice of each segmentation mask. From these measurements, live and dead bacterial ratios were calculated. (iii) In analysis of mixed biofilms, the fluorescent signal of each strain was separately processed following the same image-processing pipeline described above. In this case, the total biofilm volume was first calculated, and a slice-by-slice analysis of the resulting biofilm segmentation mask was then performed to obtain a Z-histogram showing the contribution, for each strain separately, of each slice to the total biofilm. This way, differences in the vertical positioning of each strain could be analyzed.

### Cell culture and quantification of bacterial invasion

A549 human alveolar basal epithelial cells (ATCC CCL-185) were maintained as described (15, 33 – 38), seeded to 1.5 × 10^5^ on 24-well plates for 32 h, and serum starved for 16 h before infection. For infection, PBS-normalized bacterial suspensions (OD_600_ = 1, ~10^9^ CFU/mL) were prepared by using NTHi strains freshly grown on PVX agar or sHTM agar containing Erm_11_ (stationary phase grown bacteria). Alternatively, two to five colonies were inoculated in 10-mL sBHI containing Erm_11_ and incubated for 12 h with shaking (100 rpm). Cultures were then diluted to OD_600_ of 0.07 in sBHI containing Erm_11_ and incubated at 37°C with 5% CO_2_ in agitation up to OD_600_ of 0.3 (~10^9^ CFU/mL). Two milliliters per sample were then collected and centrifuged (14,000 rcf, 5 min, RT). Pellets were resuspended in 1-mL PBS (exponentially grown bacteria). In all cases, A549 cells reached a ~90% confluence at the time of infection. To monitor bacterial invasion, a multiplicity of infection (MOI) of ~100:1 (200-µL bacterial suspension) was used for infection during 2 h in 1-mL Earle’s Balanced Salt Solution (Gibco) per well. Wells were washed three times with PBS, incubated for 1 h with RPMI 1640 containing 10% FCS, Hepes 10 mM and gentamicin (Gm) 200 µg/mL and washed again three times with PBS. Host cells were lysed with 300-µL PBS-saponin 0.025% for 10 min at RT, and serial dilutions were plated on sHTM agar. Results are expressed as log CFU/well. Bacterial infection experiments were performed in triplicate on at least three independent occasions (*n* ≥ 3).

### Live-cell imaging of bacterial infections

A549 cells were seeded to 2.5 × 10^4^ cells/well on eight-well chambered coverglass-bottomed slides for 32 h. A ~70% confluence was reached at the time of infection. Forty-five minutes before infection, the medium was replaced with 0.3-mL RPMI 1640 per well, and cells were loaded with 0.5 µM LysoTracker Deep Red (Invitrogen, L12492). GFP-expressing (86-028NP carrying pRSM2211) and mCherry-expressing (R2866 carrying pTBH04) *H. influenzae* strains were used for live imaging. Bacteria were freshly grown on sHTM agar containing Km_50_ (86-028NP carrying pRSM2211) or Erm_11_ (R2866 carrying pTBH04). Next, two to five colonies were inoculated in 10-mL sBHI (86-028NP carrying pRSM2211) or sBHI containing Erm_11_ (R2866 carrying pTBH04) and incubated for 12 h while shaking (100 rpm). Cultures were then diluted to OD_600_ of 0.07 in sBHI, containing Erm_11_ when necessary, and incubated at 37°C with 5% CO_2_ in agitation up to OD_600_ of 0.3. One milliliter per culture was then collected and centrifuged (14,000 rcf, 15 min, RT). Pellets were resuspended in RPMI 1640 and diluted to generate suspensions containing ~10^7^ CFU/mL. Ten minutes prior to imaging, the medium was replaced by 300 µL of the previously prepared suspension per well (MOI ~30:1). Bacterial concentrations were determined to prepare adequate suspensions for either single or mixed infections. The eight-well chambered slide was inserted in the incubation stage of a Zeiss LSM 800 inverted confocal microscope and incubated at 37°C and 5% CO_2_ during the entire live imaging experiment. A549 cells loaded with LysoTracker Deep Red, infected by GFP-expressing 86-028NP and/or mCherry expressing R2866 *H. influenzae* bacteria, were imaged in separate fluorescence channels at 488/510, 561/610, and 640/668 nm excitation/emission wavelengths, taking three different 2D field images per well, every 30 s for 1 h, using a Plan-Apochromat 20 × 0.8 NA objective. Time-lapse movies were analyzed using homemade scripts for Fiji. First, image drifting was corrected applying a rigid registration step using the Linear Stack Alignment with SIFT plugin for Fiji (39). Registered images were pre-processed using a median filter (r = 1) followed by a rolling ball background subtraction (r = 50). Subsequently, each channel was binarized using global intensity thresholding, obtaining separate binary masks for A549 cells stained with LysoTracker Deep Red, GFP-expressing 86-028NP, and mCherry-expressing R2866 bacteria. Using these masks, the total LysoTracker Deep Red stained area and the area fraction overlapping with GFP-expressing and mCherry expressing bacteria were calculated. Prior to further data analysis, results were normalized by the relative bacterial concentration (i.e., C_86-028NP_/C_R2866_) to account for deviations on the relative concentration of inoculum compared to the nominal 1:0, and R2866:86-028NP 1:1 and 5:1 ratios. The apparent rate at which the projected area of stained cell acidic subcellular compartments was harboring bacteria was calculated as the slope of a first-degree polynomial fitting of the infected area fraction over time. In these assays, a total of six distinct time-lapse videos were captured, per condition, over two independent well plates.

### Statistical analyses

In all cases, *P* < 0.05 value was considered statistically significant. Analyses were performed using Prism software, version 7 for Mac (GraphPad Software) statistical package, and are detailed in each figure legend.

## RESULTS

### Generation and characterization of a novel toolkit for *H. influenzae* engineering

**Fig 1:**
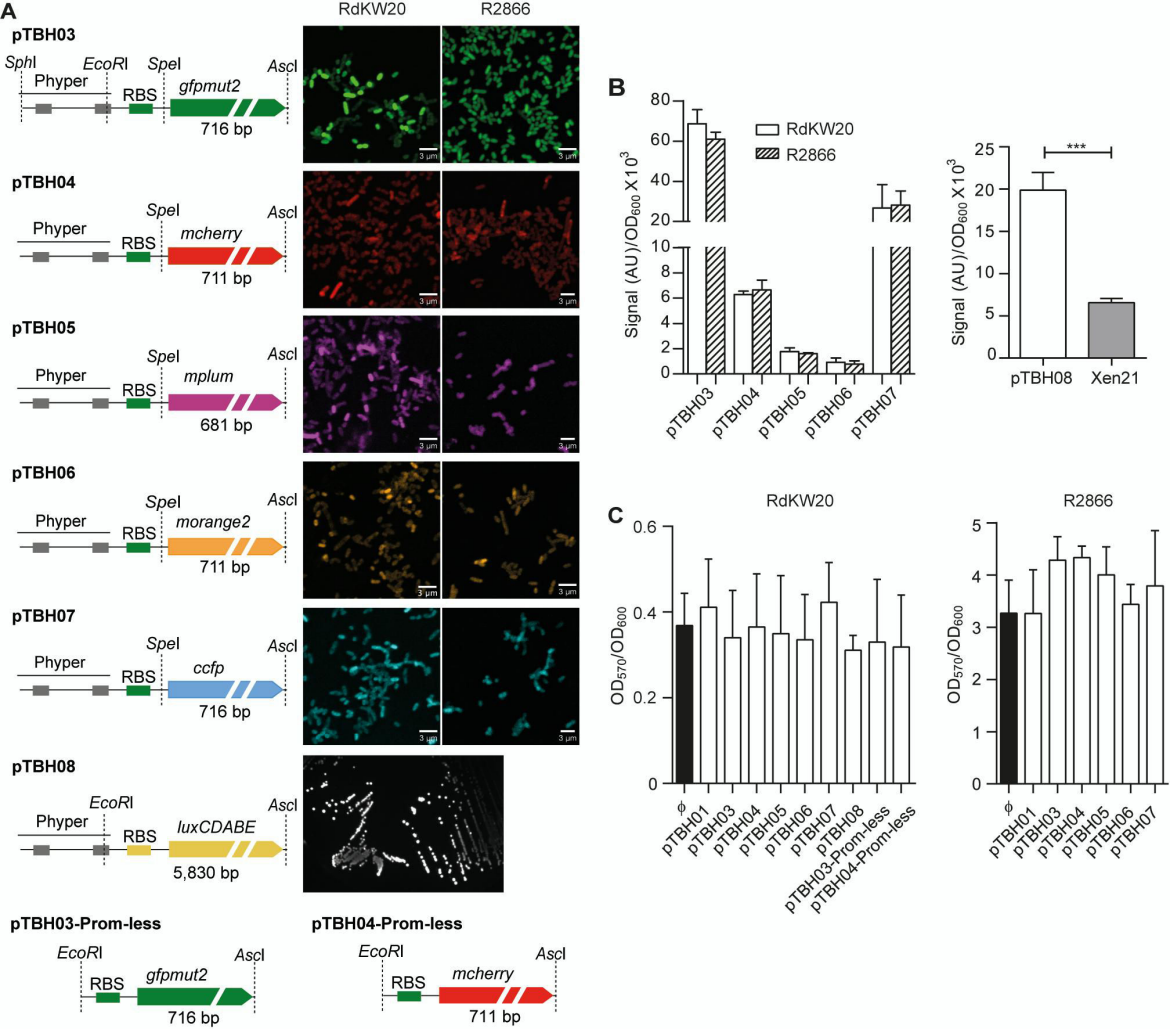
Generation of pTBH plasmid set for *H. influenzae* labeling. (**A**) Schematic representation of cargo modules in pTBH plasmids (left panels). Plasmids were introduced into RdKW20 and R2866 strains. Exponentially grown fluorescent bacteria were observed by CLSM; bioluminescence was recorded using a ChemiDoc imaging system. In all cases, representative images are shown (right panels). (**B**) Fluorescence was quantified using a SynergyH1 microplate reader; excitation wavelengths of 485, 587, 520, and 435 nm were used for GFP, mCherry, mPlum, mOrange, and cCFP, respectively (white bars, RdKW20; dashed bars, R2866). Data were obtained by dividing fluorescence arbitrary units (AUs) by bacterial growth using cultures turbidity as reference (OD_600_ = 0.3). Data are represented as mean ± SD, statistical comparisons were performed by one-way analysis of variance (ANOVA) and Bonferroni´s multiple comparison test. No significant differences were observed when comparing fluorescence between strains. Bioluminescence was quantified by using a luminescence filter cube, included in the SynergyH1 microplate reader. Results (mean ± SD) are represented as arbitrary units (AU) normalized to stationary phase-grown bacteria (OD_600_ = 0.6). Statistical comparison was performed by *t*-test (***P < 0.0001). (**C**) Biofilm growth on abiotic surfaces by *H. influenzae* RdKW20 and R2866 untransformed strains (black bars, ф) or transformed with pTBH01, pTBH03-08 (white bars), determined by crystal violet staining. Data are represented as mean ± SD; statistical comparisons were performed by one-way ANOVA and Bonferroni’s multiple comparison test. No significant differences were observed.

We generated a genetic toolbox for engineering *H. influenzae* consisting of a plasmid set named toolbox for *Haemophilus* (Table 1; Fig. S1; Fig. 1A). The pTBH structure contains a cargo module, two selectable markers, and a replication origin, flanked by restriction enzyme sites facilitating their exchange. We used a previously generated backbone plasmid suitable for *H. influenzae* transformation, named pTBH01 (15). pTBH01 contains a cargo module (1:403 bp) which includes multicloning site (MCS) and transcription terminator sequences, amplified from pCN47 (26); an erythromycin resistance gene (404:1,538 bp), amplified from pBSLerm (40) allowing selection in *H. influenzae*; a region containing the p15A replication origin and an ampicillin resistance gene (1,538:3,742 bp) excised from pACYC177 (New England Biolabs), allowing replication in *E. coli* and *H. influenzae*, and selection in *E. coli*. Such fragments were assembled by using the *Nhe*I, *Nar*I, and *Xho*I restriction sites (Fig. S1). By following the design summarized in Fig. S1, pTBH02 was generated by cloning a *Sph*I-*Asc*I fragment excised from pHRG (27) containing the Phyper constitutive promoter, a ribosomal binding site (RBS) from Gram-positive bacteria and the *gfpmut2* gene, encoding the GFP, into the pTBH01 MCS. Next, pTBH03-Pr*hmw* was generated by *Sph*I-*Spe*I cloning the *hmw* promoter and an RBS from Gram-negative bacteria into pTBH02 (15). Pr*hmw* was then exchanged by Phyper (*Sph*I and *EcoR*I flanking sites) to generate pTBH03, where we standardized the cargo module by using *Sph*I, *EcoR*I, *Spe*I, and *Asc*I restriction sites to compartmentalize promoter, RBS, and open reading frame (ORF) regions, respectively (Fig. 1A). pTBH03 was the starting point for the subsequent plasmid series, where the *gfpmut2* gene was exchanged with the *mcherry* (pTBH04), *mplum* (pTBH05), *morange2* (pTBH06), or *ccfp* (pTBH07) genes, respectively (Fig. 1A). In addition, aiming to provide suitable tools for trapping and/or exchange promoters, pTBH03 and pTBH04 derivatives were generated by *EcoR*I-*Asc*I cloning of the *gfpmut2* or *mcherry* genes into pTBH01 to generate pTBH03-Prom-less and pTBH04-Prom-less. Lastly, pTBH08 was generated by *EcoR*I-*Asc*I cloning of the RBS and the *luxCDABE* operon amplified from pSEVA236 (Standard European Vector Architecture, https://seva-plasmids.com) (Fig. 1A).

pTBH plasmids were introduced by electroporation in *H. influenzae* RdKW20 and selected using Erm_11_. In general terms, planktonic growth of the engineered strains was comparable to the untransformed strain (Fig. S2A left panel). Exponentially grown bacterial suspensions (OD_600_ = 0.3) were collected, and fluorescence or bioluminescence was quantified accordingly (Fig. 1B). When using RdKW20-pTBH08, bioluminescence was significantly higher than that by the RdKW20 Xen21 strain [RdKW20 derivative with a *luxCDABE* chromosome insert; Table S1 (41)]. Next, crystal violet staining showed that RdKW20 biofilm growth on abiotic surfaces was maintained upon plasmid transformation (Fig. 1C left panel).

To expand the range of *H. influenzae* hosts and generate tools for assessing polyclonal infections by phenotypically divergent strains, we next screened strains amenable for pTBH transformation in an available collection of clinical isolates. A total of 31 additional strains were used from which nine were successfully transformed with at least one pTBH plasmid, including NTHi R2866 and 86-028NP otitis media isolates (42, 43), and the NTHi P589, P593, P597, P607, P610, P621, and P656 COPD isolates (16) (Table S1; Fig. S3). Given that the methylation profile of plasmid DNA may account for difficulties in terms of transformation efficiency, we purified pTBH03 from RdKW20 and used it to re-transform RdKW20 itself, which, indeed, increased transformation efficiency from ~3 × 10^−8^ when using pTBH03 purified from *E. coli*, to ~3 × 10^−6^ when using pTBH03 purified from RdKW20. Notably, transformation of NTHi 86-028NP was only successful when using pTBH03 purified from RdKW20.

R2866 was transformed with the complete pTBH plasmid series. Biofilm growth on this strain was higher than the RdKW20 strain, and it was selected for further study together with RdKW20. Fluorescence of R2866 derivative strains was comparable to that of their corresponding RdKW20 engineered strains (Fig. 1B left panel). R2866 transformants showed planktonic and biofilm growth that were comparable to those of the untransformed strain (Fig. S2A; Fig. 1C right panels, respectively).

**Fig 2:**
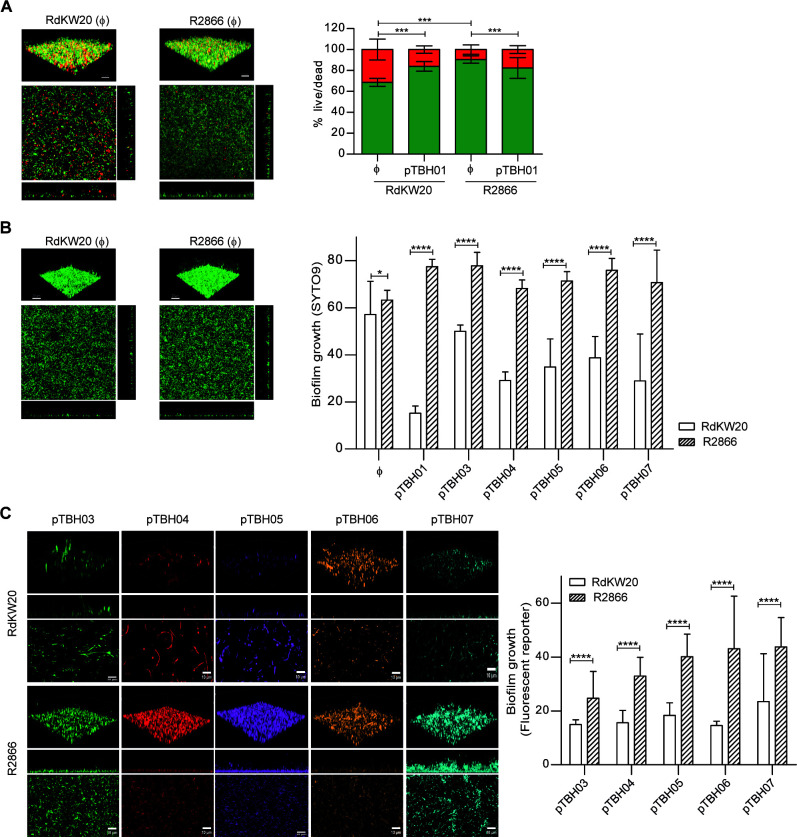
Biofilm growth by *H. influenzae*-pTBH derivative strains determined by CLSM. (**A**) Bacterial viability determination by Live-Dead staining: for each strain, upper panels show 3D renderings of the biofilms using Imaris software; lower panels show XZ- and YZ-plane lateral and XY axial projections of the confocal image from the densest slice from each biofilm. Bacterial live (green bar)/dead (red bar) percentages were calculated as described in the Methods section. Data obtained for RdKW20 and R2866 untransformed (Φ), and -pTBH01 transformed strains, are shown. Representative images are shown for untransformed strains. Data are represented as mean ± SD; statistical comparisons were performed by one-way ANOVA and Tukey‘s multiple comparison test. Significant differences were found when comparing RdKW20 (Φ) to RdKW20-pTBH01, *P* < 0.0001; R2866 (Φ) to R2866-pTBH01, *P* < 0.001; RdKW20 (Φ) to R2866 (Φ), *P* < 0.0001. (**B**) Biofilm growth analysis by quantification of SYTO9: RdKW20 and R2866 untransformed (Φ), and pTBH-transformed biofilms were stained with SYTO9. Representative images are shown for untransformed strains. Upper panels show 3D renderings of the biofilms using Imaris software; lower panels show XZ- and YZ-plane lateral and XY axial projections of the confocal image from the densest slice from each biofilm. Biofilm growth (shown as %) was quantified as the relative area occupied by SYTO9 fluorescence in the densest slice of the biofilm image stacks (see Methods section). For each strain pair, i.e., RdKW20 versus R2866, statistical comparisons of means were performed by Mann-Whitney multiple comparison test; significant differences were observed in all cases (*P < 0.05, ****P < 0.0001). For each bacterial strain, i.e., RdKW20 Φ and derivatives; R2866 Φ and derivatives, statistical comparisons of means were performed by one-way ANOVA and Kruskal-Wallis comparison test. For RdKW20, fluorescence was lower for all transformed derivative strains compared to RdKW20 (Φ), except for RdKW20-pTBH03. For R2866, no significant differences were observed. (**C**) Biofilm growth analysis by quantification of fluorescence emitted by pTBH03-07 transformed bacterial strains: upper panels show 3D renderings of the biofilms using the Imaris software; middle and lower panels show XZ-plane lateral and XY axial projections of the confocal z-stack images of the same data sets. Representative images are shown in all cases. Fluorescent biofilm growth (shown as %) was quantified as the relative area occupied by each fluorescent signal in the densest slice of the biofilm image stacks (see Methods section). For each strain pair, i.e., RdKW20-pTBH03 versus R2866-pTBH03, etc., statistical comparisons of means were performed by Mann-Whitney multiple comparison test. Significant differences were observed in all cases, ****P < 0.0001. For each bacterial strain, i.e., RdKW20-pTBH03 to 07 and R2866-pTBH03 to 07, statistical comparisons of means were performed by one-way ANOVA and Kruskal-Wallis comparison test. No differences were observed.

Next, we assessed bacterial viability after biofilm growth. Live-Dead staining was used for RdKW20 and R2866 untransformed (Φ) and carrying the pTBH backbone, i.e., RdKW20-pTBH01 and R2866-pTBH01. Such staining could not be used for strains transformed with the set of pTBH plasmids as there is severe overlap between the fluorescence emission spectra of most plasmids and the dead marker (propidium iodide), which could strongly compromise the interpretation of the results. Viability numbers for both RdKW20 and R2866 strains ranged from 61% to 96%, being higher for R2866; upon pTBH01 transformation, slight bacterial viability differences were found for both strains (Fig. 2A).

We next compared the capacity to form biofilm by all the strains. To do so, biofilm growth was quantified separately from both SYTO9 staining and from the fluorescence emitted by each reporter protein. Fig. 2B shows the results of quantifying total biofilm growth based on SYTO9 staining. R2866 biofilm growth was comparable among untransformed (Φ) and all transformed strain derivatives and higher than that of RdKW20 in all cases. Differently, quantification of RdKW20 biofilm growth decreased upon pTBH plasmid transformation, as fluorescence was lower for all transformed derivative strains compared to RdKW20 (Φ), except for RdKW20-pTBH03. Fig. 2C shows the results of quantifying biofilm growth based on the fluorescence emitted by bacteria transformed with the pTBH03 to 07 plasmids, i.e., GFP, Cherry, Plum, Orange, and CFP, respectively. R2866 biofilm growth was in all cases higher than that of the respective RdKW20 strains; R2866 and RdKW20 biofilm growth was comparable among each respective panel of transformed strain derivatives. In all cases, quantification of SYTO9 signal rendered higher numbers than that determined upon quantification of the fluorescent proteins (Fig. 2B and C, right panels).

Lastly, we assessed pTBH01 backbone stability. To do so, cultures of *H. influenzae* RdKW20-pTBH01 and R2866-pTBH01 were inoculated and propagated every 12 h for eight serial passages in medium without antibiotics. The rate of colonies that remained resistant to Erm 11 µg/mL after each subculture slightly decreased and then stayed stable for R2866 (~80%) and decreased to ~40% for RdKW20 (Fig. S2B).

Overall, a panel of plasmids suitable to engineer fluorescent or bioluminescent *H. influenzae* was generated and introduced in a collection of independent strains. Plasmid stability required keeping antibiotic pressure over time, and both conditions and duration should be carefully considered in the planning phase of each assay.

### Architecture of *H. influenzae* polyclonal biofilms under static culture conditions

**Fig 3:**
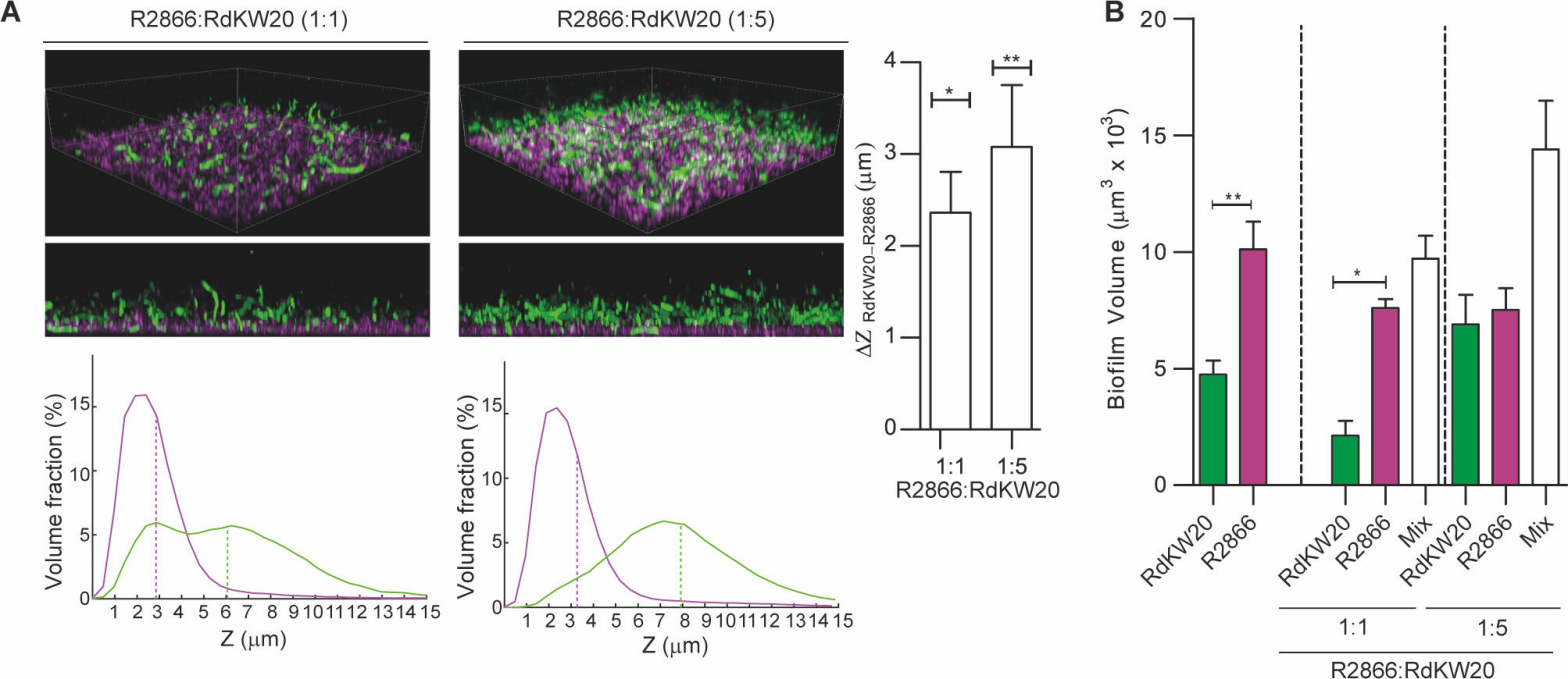
Mixed biofilm growth by differentially labeled fluorescent *H. influenzae* strains. RdKW20-pTBH03 and R2866-pTBH05 were used as representative strains, shown in green and magenta, respectively. (**A**) Two different views of a 3D render of the mixed biofilm with R2866:RdKW20 1:1 (left) or 1:5 (right) ratios are shown at the top, and the corresponding Z-histograms showing the displacement in Z position between strains are shown at the bottom. Right panel: measured difference between the center of mass in the Z axis of the two strains to quantify the relative displacement between strains in the Z axis. Z-displacements were statistically tested (against the null hypothesis, i.e., no relative displacement between strains) using one-tail *t*-test (*P < 0.05, **P < 0.01). (**B**) Individual contribution of each strain (green bars, RdKW20; magenta bars, R2866) and of both strains combined (white bars, mixed) to the volume of mixed biofilms. Pairwise comparisons between the individual contributions of RdKW20 and R2866 were performed using two-tailed *t*-tests (*P < 0.05, **P < 0.01).

We next assessed the architecture and relative distribution of phenotypically divergent *H. influenzae* strains when coexisting in polyclonal biofilms. We used R2866-pTBH05 and RdKW20-pTBH03, as representative strains with different biofilm growth capacity (Fig. 2 and 3). For mixed biofilm growth, two mixed ratios were used as starting inoculum, R2866:RdKW20 1:1 or 1:5. We selected bacteria bearing pTBH05 and pTBH03 based on the good spectral separation of the encoded fluorescent protein pairs, i.e., mPlum in pTBH05 and GFPmut2 in pTBH03, which enabled simultaneous unequivocal identification of each strain in the mixed biofilms. In all cases, R2866 occupied the abiotic surface forming a dense layer. In contrast, RdKW20 barely bound to the surface but seemed to use the R2866 biofilm as a scaffold for attachment. Quantification of each bacterial volume fraction along the Z-axis supported a bilayer architecture, which became clearer when the RdKW20 strain proportion was increased in the starting inoculum (1:5) (Fig. 3A). Relative contribution of each strain to the biofilm volume was also measured. When mixed at 1:1 ratio, R2866 contribution was significantly higher than that of RdKW20; however, both strains contributed in a similar manner when the RdKW20 proportion was increased in the starting inoculum (1:5). Under the later conditions, biofilm volume was higher than when using a 1:1 starting inoculum (Fig. 3B).

In summary, *H. influenzae* polyclonal biofilm growth was modeled by co-culturing the R2866 and RdKW20 strains under static conditions, with a bilayer organization supporting the notion of R2866 acting as a scaffold for RdKW20, where each strain contribution to the biofilm volume was dependent on their respective proportion at the assay starting point.

### Differential antibiotic efficacy on *H. influenzae* polyclonal biofilms

**Fig 4:**
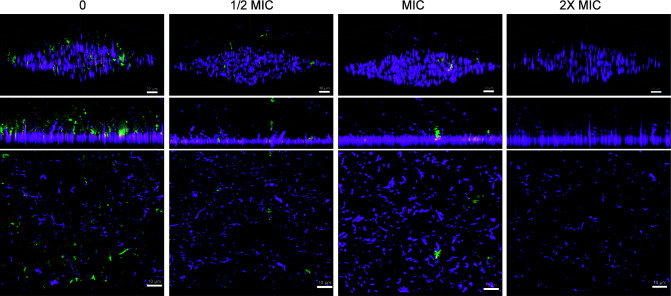
Selective effects of ampicillin on mixed biofilm growth by differentially labeled fluorescent *H. influenzae* strains. RdKW20-pTBH03 and R2866-pTBH05 were used as representative strains. MIC for ampicillin: 32 µg/mL for RdKW20-pTBH03; >256 µg/mL for R2866-pTBH05. Biofilm growth was acquired by CLSM in the absence (0) or in the presence of ampicillin, used at 16, 32, or 64 µg/mL, corresponding to ½ MIC, MIC, or 2× MIC for the RdKW20-pTBH03 strain. The top panels show representative 3D renderings by Imaris software; middle and bottom panels show XZ lateral and XY axial projections of the corresponding image stacks.

Biofilm formation is a leading cause of antibiotic failure as biofilms are environmental reservoirs of resistant bacteria and biofilm infections are extremely challenging to treat (44). This may pose a notable challenge in biofilms where strains with significantly different MIC for the antibiotic of choice coexist. We addressed this issue by testing the inhibitory effect of an antibiotic with significantly different MIC values between the RdKW20 and R2866 strains, on the formation of mixed biofilms. Cefuroxime, cefepime, ceftriaxone, imipenem, meropenem, chloramphenicol, tetracycline, azithromycin, rifampicin, nalidixic acid, or levofloxacin could not be considered because both strains had comparable MIC values (Table S3). Ampicillin was the antibiotic of choice to test its inhibitory effect on the formation of mixed biofilms by R2866-pTBH05 and RdKW20-pTBH03 strains. pTBH plasmids have an ampicillin resistance gene, but we experimentally determined that MIC for ampicillin differs between RdKW20 and R2866 pTBH-transformed strains, being 32 µg/mL for RdKW20-pTBH03 and >256 µg/mL for R2866-pTBH05. Ampicillin 16, 32, and 64 µg/mL effect was tested, i.e., RdKW20-pTBH03 ½ MIC, MIC, and 2× MIC. A dose-dependent inhibitory effect was observed on RdKW20 but not on R2866 biofilm growth, which remained unaltered (Fig. 4). Therefore, under mixed biofilm growth conditions, antibiotics may act selectively and discriminate between *H. influenzae* strains depending on their respective susceptibility level.

### Dynamics of *H. influenzae* polyclonal airway epithelial cell co-infection

**Fig 5:**
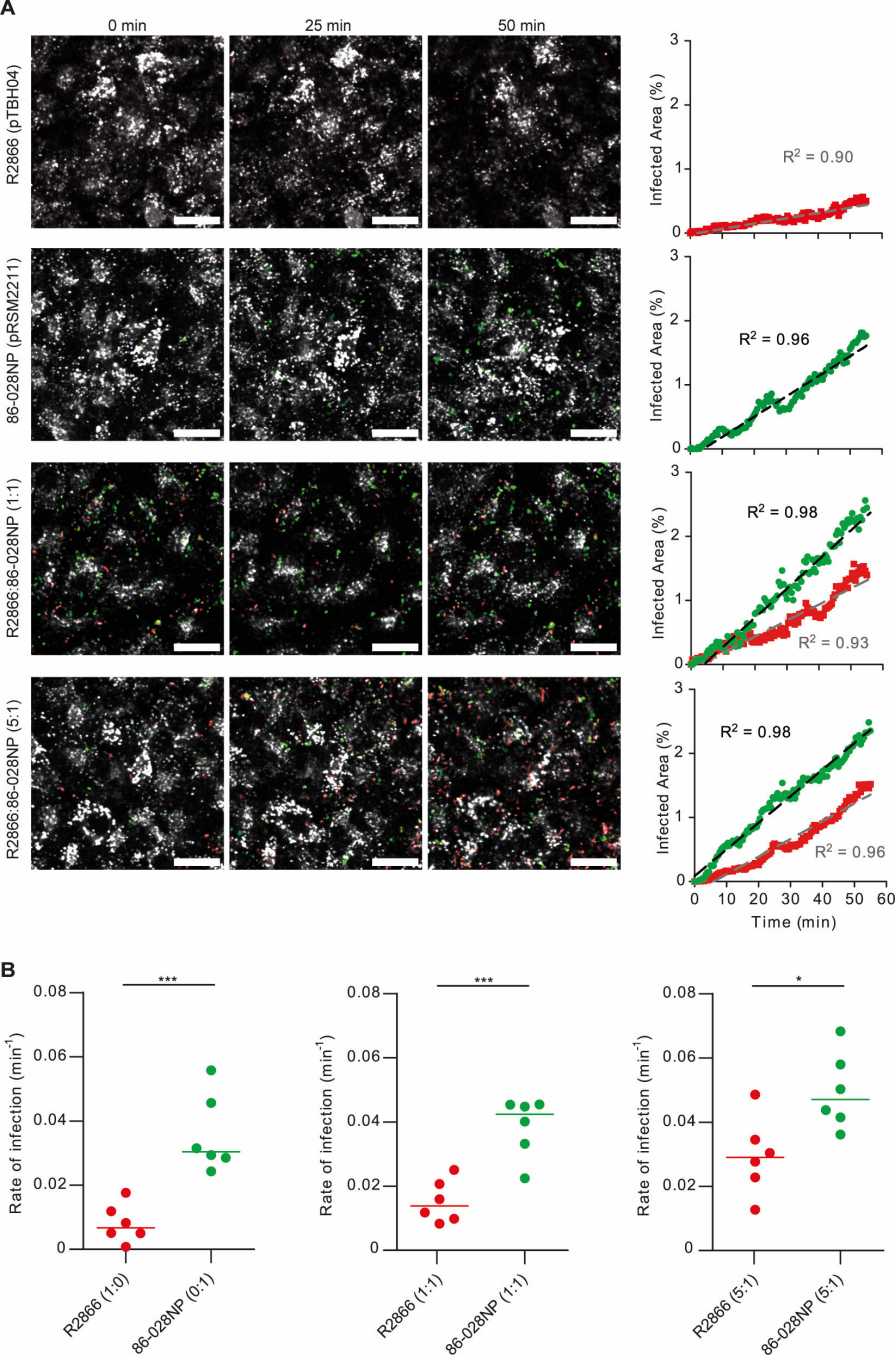
Dynamics of airway epithelial cell co-infection by differentially labeled fluorescent *H. influenzae* strains. Time-lapse CLSM microscopy of living A549 cells infected by *H. influenzae* R2866-pTBH04 (red, panel 1), 86-028NP-pRSM2211 (green, panel 2), or co-infected by R2866:86-028NP, ratios 1:1 or 5:1 (panels 3 and 4, respectively). (**A**) Representative images from time-lapse imaging of A549 cells loaded with LysoTracker (white) and infected. The imaging starting point, referred to as 0 min, was initiated at 10 min post-infection. Cells were imaged every 30 s until 60 min after infection. Bacteria are seen to co-localize with LysoTracker, and the area of co-localization was measured. The elapsed time is shown on the top of each image (for videos, see Movies S1 through S4). On the right, graphs showing the average dynamics of infection or co-infection of the strains, quantified as the normalized overlap between the bacterial bodies and the LysoTracker stained cell compartments. Scale bar: 20 microns. (**B**) Pairwise comparisons between R2866 and 86–028NP infection rates were performed using two-tailed *t*-tests (*P < 0.05, ***P < 0.001).

Besides the ability to grow as biofilms, another *H. influenzae* lifestyle contributing to persistence is the ability to occupy subcellular acidic compartments within airway epithelial cells (45, 46). Significant heterogeneity among strains is observed in terms of epithelial invasion, from highly invasive strains such as 86-028NP, P651, or P652 (16, 47), to those that are two to three orders of magnitude less invasive, such as RdKW20 or R2866 (Fig. S4). Fluorescent labeling did not modify the bacterial ability to invade airway epithelial cells, as shown for 86-028NP-pRSM2211 (25) and for RdKW20 and R2866 when transformed with the pTBH series. Besides, epithelial invasion by selected *H. influenzae* strains was comparable when using exponential or stationary grown bacteria upon MOI maintenance (Fig. S4B). Next, we assessed the dynamics of bacterial cell invasion by phenotypically different *H. influenzae* strains when co-infecting airway epithelial cells by using live confocal microscopy. We selected highly invasive 86-028NP-pRSM211 and less invasive R2866-pTBH04 strains to infect A549 cells in single-strain or co-infection experiments. In the later ones, two mixed inoculum ratios were used, either R2866:86-028NP 1:1 or 5:1. Representative time-lapse sequences are shown in Movies S1 to S4. Bacterial co-localization with LysoTracker was used as bacterial intracellular location readout to derive the percentage of A549 infected area, corrected by the relative concentration between 86-028NP and R2866 (Fig. 5A). Time-lapse imaging showed that, under our experimental conditions, co-localization of fluorescently labeled *H. influenzae* strains with LysoTracker-stained subcellular compartments increased linearly over time. The apparent infection rate, as displayed in Fig. 5B, provides a quantitative measure of each strain intrinsic ability to localize within acidic endosomes over time. When co-infecting at the 1:1 ratio, 86-028NP demonstrated a higher infection rate compared to the R2866 strain. At 5:1 ratio, the rate of infection of R2866 was higher, but still significantly lower than that of 86-028NP.

In sum, we developed tools suitable to analyze the dynamics of *H. influenzae* intracellular location allowing us to differentiate between clinical isolates with different epithelial invasiveness.

## DISCUSSION

In this work, we addressed the study of polyclonality, i.e., the dynamics of multiple coexisting lineages from the same bacterial pathogen species. This is a relevant but unattended issue as, for practical reasons, standardized clinical procedures may not always involve separate collection of multiple independent colonies after pathogen identification, likely leading to antibiotic susceptibility profiling of the most abundant lineage present in the clinical specimen. Although informative for correct decision making in most cases, heterogeneity in antibiotic susceptibility among coexisting clones may undermine resistance and lead to therapeutic failure. This may be the case for *H. influenzae*, where multiple strains coexist in diseased and healthy individuals (4 – 7, 17, 18), displaying differences in susceptibility for a variety of antibiotics (4). Indeed, genomic diversity is a hallmark of nontypeable *H. influenzae* strains, as competence in acquiring genomic material facilitates genomic plasticity, leading to an open pan-genome with a large accessory genome (48). Such diversity confers phenotypic heterogeneity, among others, in terms of natural transformation frequency and airway epithelial cell invasion (47, 49). Despite reported coexisting lineages and heterogeneity, a drawback to improve our understanding of the *H. influenzae* host-pathogen interplay is the lack of genetic tools for strain engineering, as reports on effective shuttle vectors are scarce (50). Here, we present a novel set of plasmids contributing to overcome this gap. pTBH plasmids were designed with a modular architecture using the SEVA concept as a model (22) (not following the SEVA rules themselves), and this versatile scaffold was built on already available pTBH01, pTBH02, and pTBH03-Pr*hmw* plasmids (15). Although these tools are of great potential usefulness, some considerations are next discussed. (i) p15A replication origin was previously used in *H. influenzae* (51, 52), but a strong strain bias regarding transformation efficacy was observed. Here, electroporation was attempted in 32 different *H. influenzae* strains, and transformants were obtained in 10 of them. Even more, these plasmids may be of use for other bacteria as, for example, transformants were nicely obtained in the nonrelated Gram-negative species *Salmonella typhimurium* (Fig. S3, bottom panel). For practical reasons, the set of pTBH plasmids was purified from *E. coli* because purification yields were higher than those obtained upon pTBH purification from RdKW20. However, despite low yield, plasmid purification from a recipient *H. influenzae* strain may be advantageous as our results support it as a helpful strategy to expand the range of recipient strains and/or increase transformation efficiency. (ii) The pJMA-1 plasmid, naturally found in *H. parasuis* and bearing p15A replication origin, was highly stable in RdKW20 (30). Although sharing the same replication origin, pTBH stability seems to be lower and variable between strains likely limiting assay duration in the absence of antibiotic in a strain-dependent manner. Our biofilm formation assays were performed in the presence of antibiotic, and, if antibiotic is present, selective pressure should contribute keeping the plasmid during the entire experiment, although assays involving bacterial growth may be influenced by strain-dependent plasmid maintenance. These aspects should be considered in future work. (iii) pTBH aims modularity and versatility, but it may be also advantageous in terms of signal as, when using RdKW20-pTBH08, bioluminescence was higher than in the RdKW20 Xen21 strain, maybe related to differences in copy number and/or promoter strength. (iv) SYTO9 staining showed comparable R2866 biofilm growth among untransformed (Φ) and transformed strain derivatives, but RdKW20 biofilm growth decreased in pTBH-containing derivative strains. This was unexpected as crystal violet staining showed comparable biofilm growth for each strain panel, i.e., did not show intra-strain differences (Fig. 1C and 2B). Assay setting and duration were comparable, but crystal violet staining involves multiple washing steps that could contribute detaching bacteria not firmly attached to the substrate, which may be particularly critical for RdKW20. This issue should be always considered and carefully analyzed when using pTBH plasmids in other bacterial strains and/or assay types. (v) For both RdKW20 and R2866 derivative strains, biofilm growth differed when quantifying SYTO9 or the GFP, Cherry, Plum, Orange, and CFP fluorescent proteins. This was also unexpected as all assays were performed in the presence of antibiotic for plasmid maintenance and also considering that the algorithms used to quantify biofilm growth are not affected by differences in the fluorescence intensity. However, we cannot exclude that, in some cases, a portion of the existing biofilm does not express the expected fluorescence or that some bacteria have low fluorescence quantum yield, displaying fluorescence emission below the limit of detection of the microscope used.

As a proof of principle, pTBH plasmids were used to address two *H*. *influenzae* lifestyles contributing to persistence, in a minimized polyclonal set up consisting of two co-existing heterogeneous strains. To our knowledge, this is the first report of this kind, contingent on strain and assay conditions, and paves the way for further analyses of more complex mixed settings such as biofilm growth under flow conditions and/or on biotic surfaces, infection of biological systems with higher complexity than 2D cultured cells, etc. Also, the observed mixed biofilm growth architecture, where strains showed heterogeneous behavior, led us to speculate that poor biofilm formers may benefit from better biofilm formers upon mixed infection. Future work will assess other strain pairs to address this issue. Notably, ampicillin selective effects on mixed biofilms were clear, bringing us on track to carefully evaluate the fine-tuned balance of antibiotic therapeutics in clinical specimens suspected of polyclonal infection. On the other hand, epithelial co-infection dynamics by mixing a previously described hyperinvasive strain [86-028NP, (47)] with another strain, R2866, which invades epithelial cells at a lower rate, suggested that once bacterial cells adhere to the epithelial cell surface, they may enter rapidly as co-localization with LysoTracker-loaded compartments was seen from 10 min onwards. Also, R2866 infection rate seemed to be enhanced by the presence of the co-infecting 86-028NP strain. We cannot provide an explanation for this trend, and these observations will be pursued in future work.

In summary, pTBH is a novel set of plasmids with a modular architecture compatible with *H. influenzae* requirements. The suitability of this plasmid series is not restricted to *H. influenzae*, so it can be used in other bacterial species. Importantly, it is necessary to take into account that appropriate phenotyping controls need to be always made for all newly transformed strains. Detailed experimental and quantitative image analysis methods, together with proof-of-principle examples, are presented to show the ample possibilities of 3D microscopy, combined with quantitative image analysis, to unravel the co-habitation and co-infection dynamics of fluorescently labeled bacterial strains. Modeling of *H. influenzae* polyclonal infection lifestyles prompts to further pTBH plasmid development, improvement, and application to generate useful information in clinically relevant settings.

## SUPPLEMENTAL MATERIAL

Supplemental material spectrum.00993-23-s0001.pdf Tables S1 to S3 and Fig. S1 to S4.

Movie S1 spectrum.00993-23-s0002.mp4 Representative time-lapse sequence for A549 cell infection by *H. influenzae* R2866.

Movie S2 spectrum.00993-23-s0003.mp4 Representative time-lapse sequence for A549 cell infection by *H. influenzae* 86-028NP.

Movie S3 spectrum.00993-23-s0004.mp4 Representative time-lapse sequence for A549 cell co-infection by *H. influenzae* R2866:86-028NP, ratio 1:1.

Movie S4 spectrum.00993-23-s0005.mp4 Representative time-lapse sequence for A549 cell co-infection by *H. influenzae* R2866:86-028NP, ratio 5:1.

ASM does not own the copyrights to Supplemental Material that may be linked to, or accessed through, an article. The authors have granted ASM a non-exclusive, world-wide license to publish the Supplemental Material files. Please contact the corresponding author directly for reuse.
