# Supplementary material for: In vitro modeling of polyclonal infection dynamics within the human airways by Haemophilus influenzae differential fluorescent labeling
Source: Microbiol Spectr. 2023 Oct 5;11(6):e00993-23. doi: 10.1128/spectrum.00993-23 (PMC10714817; doi:10.1128/spectrum.00993-23)
Supplement: Supplemental material — Tables S1 to S3 and Fig. S1 to S4. [file spectrum.00993-23-s0001.pdf]

Supplemental Material

In vitro modeling of polyclonal infection dynamics within the human airways by *Haemophilus influenzae* differential fluorescent labelling

Beatriz Rapún-Araiz[1,2+], Ioritz Sorzabal-Bellido[3+], Javier Asensio-López[1,2,4], María Lázaro-Díez[1,2], Mikel Ariz[3], Carlos Sobejano de la Merced[3], Begoña Euba[1,2], Ariadna Fernández-Calvet[1], Ivan Cortés-Domínguez[3], Saioa Burgui[4], Alejandro Toledo-Arana[1], Carlos Ortiz-de-Solórzano[3,5,6], Junkal Garmendia[1,2,7#]

[1]Instituto de Agrobiotecnología, Consejo Superior de Investigaciones Científicas (IdAB-CSIC)-Gobierno de Navarra, Mutilva, Spain; [2]Conexión Nanomedicina CSIC (NanomedSIC); [3]Laboratorio de Sistemas Microfisiológicos y Biología Cuantitativa, Programa de Ingeniería Biomédica, Centro de Investigación Médica Aplicada (CIMA), Pamplona, Spain; [4]Asociación de la Industria Navarra (AIN)-Gobierno de Navarra, Cordovilla, Spain; [5]Centro de Investigación Biomédica en Red de Enfermedades Oncológicas (CIBERONC), Madrid, Spain; [6]Instituto de Investigación Sanitaria de Navarra (IdiSNA), Pamplona, Spain; [7]Centro de Investigación Biomédica en Red de Enfermedades Respiratorias (CIBERES), Madrid, Spain

Content:

Table S1. Bacterial strains used in this study.

Table S2. List of primers used in this study.

Table S3. Antibiotic minimal inhibitory concentration (MIC).

Figure S1. Schematic representation of pTBH plasmid architecture.

Figure S2. *H. influenzae* planktonic growth and plasmid stability assays.

Figure S3. *H. influenzae* biofilm growth on abiotic surfaces.

Figure S4. Invasion of A549 type II pneumocytes by *H. influenzae* strains.

Movie S1. Representative time-lapse sequence for A549 cell infection by *H. influenzae* R2866.

Movie S2. Representative time-lapse sequence for A549 cell infection by *H. influenzae* 86-028NP.

Movie S3. Representative time-lapse sequence for A549 cell co-infection by *H. influenzae* R2866:86-028NP, ratio 1:1.

Movie S4. Representative time-lapse sequence for A549 cell co-infection by *H. influenzae* R2866:86-028NP, ratio 5:1.

Table S1. Bacterial strains used in this study.

Strains	Description	Source
E. coli		
TOP10/P350	Cloning strain. F- *mcrA* Δ(*mrr-hsd*RMS-*mcr*BC) Φ80*lac*ZΔM15 Δ*lac*X74 recA1 *ara*D139 Δ(araleu)7697 *gal*U *gal*K *rps*L (StrR) *end*A1 *nup*G	Fisher Scientific
H. influenzae		
86-028NP/P560	Otitis media clinical isolate	(1)
86-028NP (pRSM2211)/P580	86-028NP derivative, transformed with pRSM2211	(2)
RdKW20/P189	Laboratory strain, capsule-deficient serotype d	(3)
RdKW20 Xen21/P871	RdKW20 derivative with a *luxCDABE* insert	(4)
R2866/P570	Blood isolate, from invasive disease	(5)
RdKW20 (pTBH01)/1085	RdKW20 derivative, transformed with pTBH01, a pACYC177 derivative containing an Erm^R gene and a MCS. Amp^R, Erm^R	(6)
RdKW20 (pTBH03)/1088	RdKW20 derivative, transformed with pTBH03, carrying the *gfpmut2* gene expressed under the constitutive promoter Phyper. Amp^R, Erm^R	This study
RdKW20 pTBH03-Prom-less/1262	RdKW20 derivative, transformed with pTBH03-Prom-less, a pTBH01 derivative containing the *gfpmut2* gene without promoter. Amp^R, Erm^R	This study
RdKW20 (pTBH04)/1263	RdKW20 derivative, transformed with pTBH04, carrying the *mcherry* gene expressed under the constitutive promoter Phyper. Amp^R, Erm^R	This study
RdKW20 pTBH04-Prom-less/1264	RdKW20 derivative, transformed with pTBH04-Prom-less, a pTBH01 derivative containing the *mcherry* gene without promoter. Amp^R, Erm^R	This study
RdKW20 (pTBH05)/1265	RdKW20 derivative, transformed with pTBH05, carrying the *mplum* gene expressed under the constitutive promoter Phyper. Amp^R, Erm^R	This study
RdKW20 (pTBH06)/1266	RdKW20 derivative, transformed with pTBH06, carrying the *morange* gene expressed under the constitutive promoter Phyper. Amp^R, Erm^R	This study
RdKW20 (pTBH07)/1267	RdKW20 derivative, transformed with pTBH07, carrying the *ccfp* gene expressed under the constitutive promoter Phyper. Amp^R, Erm^R	This study
RdKW20 (pTBH08)/1383	RdKW20 derivative, transformed with pTBH08, carrying the *luxCDABE* operon expressed under the constitutive promoter Phyper. Amp^R, Erm^R	This study
R2866 (pTBH01)/1269	R2866 derivative, transformed with pTBH01, a pACYC177 derivative containing an Erm^R gene and a MCS. Amp^R, Erm^R	This study
R2866 (pTBH03)/1270	R2866 derivative, transformed with pTBH03, carrying the *gfpmut2* gene expressed under the constitutive promoter Phyper. Amp^R, Erm^R	This study
R2866 (pTBH03)/1270	R2866 derivative, transformed with pTBH03, carrying the *gfpmut2* gene expressed under the constitutive promoter Phyper. Amp^R, Erm^R	This study
R2866 (pTBH04)/1275	R2866 derivative, transformed with pTBH04, carrying the *mcherry* gene expressed under the constitutive promoter Phyper. Amp^R, Erm^R	This study
R2866 (pTBH05)/1278	R2866 derivative, transformed with pTBH05, carrying the *mplum* gene expressed under the constitutive promoter Phyper. Amp^R, Erm^R	This study
R2866 (pTBH06)/1280	R2866 derivative, transformed with pTBH06, carrying the *morange* gene expressed under the constitutive promoter Phyper. Amp^R, Erm^R	This study
R2866 (pTBH07)/1282	R2866 derivative, transformed with pTBH07, carrying the *ccfp* gene expressed under the constitutive promoter Phyper. Amp^R, Erm^R	This study
P589 (pTBH03)/1442	P589 derivative, transformed with pTBH03, carrying the *gfpmut2* gene expressed under the constitutive promoter Phyper. Amp^R, Erm^R	This study
P589 (pTBH05)/1443	P589 derivative, transformed with pTBH05, carrying the *mplum* gene expressed under the constitutive promoter Phyper. Amp^R, Erm^R	This study

P597 (pTBH03)/1444	P597 derivative, transformed with pTBH03, carrying the *gfpmut2* gene expressed under the constitutive promoter Phyper. Amp^{R}, Erm^{R}	This study
P597 (pTBH05)/1445	P597 derivative, transformed with pTBH05, carrying the *mplum* gene expressed under the constitutive promoter Phyper. Amp^{R}, Erm^{R}	This study
P610 (pTBH03)/1446	P610 derivative, transformed with pTBH03, carrying the *gfpmut2* gene expressed under the constitutive promoter Phyper. Amp^{R}, Erm^{R}	This study
P610 (pTBH05)/1447	P610 derivative, transformed with pTBH05, carrying the *mplum* gene expressed under the constitutive promoter Phyper. Amp^{R}, Erm^{R}	This study
P607 (pTBH03)/1448	P607 derivative, transformed with pTBH03, carrying the *gfpmut2* gene expressed under the constitutive promoter Phyper. Amp^{R}, Erm^{R}	This study
P607 (pTBH05)/1449	P607 derivative, transformed with pTBH05, carrying the *mplum* gene expressed under the constitutive promoter Phyper. Amp^{R}, Erm^{R}	This study
P656 (pTBH03)/1450	P656 derivative, transformed with pTBH03, carrying the *gfpmut2* gene expressed under the constitutive promoter Phyper. Amp^{R}, Erm^{R}	This study
P656 (pTBH05)/1451	P656 derivative, transformed with pTBH05, carrying the *mplum* gene expressed under the constitutive promoter Phyper. Amp^{R}, Erm^{R}	This study
P593 (pTBH03)/1452	P593 derivative, transformed with pTBH03, carrying the *gfpmut2* gene expressed under the constitutive promoter Phyper. Amp^{R}, Erm^{R}	This study
P593 (pTBH05)/1453	P593 derivative, transformed with pTBH05, carrying the *mplum* gene expressed under the constitutive promoter Phyper. Amp^{R}, Erm^{R}	This study
P621 (pTBH05)/1454	P621 derivative, transformed with pTBH05, carrying the *mplum* gene expressed under the constitutive promoter Phyper. Amp^{R}, Erm^{R}	This study
P589 (pTBH04)/1456	P589 derivative, transformed with pTBH04, carrying the *mcherry* gene expressed under the constitutive promoter Phyper. Amp^{R}, Erm^{R}	This study
P589 (pTBH06)/1457	P589 derivative, transformed with pTBH06, carrying the *morange* gene expressed under the constitutive promoter Phyper. Amp^{R}, Erm^{R}	This study
P589 (pTBH07)/1458	P589 derivative, transformed with pTBH07, carrying the *ccfp* gene expressed under the constitutive promoter Phyper. Amp^{R}, Erm^{R}	This study
P593 (pTBH01)/1459	P593 derivative, transformed with pTBH01 a pACYC177 derivative containing an Erm^{R} gene and a MCS. Amp^{R}, Erm^{R}	This study
P593 (pTBH07)/1460	P593 derivative, transformed with pTBH07, carrying the *ccfp* gene expressed under the constitutive promoter Phyper. Amp^{R}, Erm^{R}	This study
P597 (pTBH01)/1461	P597 derivative, transformed with pTBH01 a pACYC177 derivative containing an Erm^{R} gene and a MCS. Amp^{R}, Erm^{R}	This study
P597 (pTBH07)/1462	P597 derivative, transformed with pTBH07, carrying the *ccfp* gene expressed under the constitutive promoter Phyper. Amp^{R}, Erm^{R}	This study
P607 (pTBH01)/1463	P607 derivative, transformed with pTBH01 a pACYC177 derivative containing an Erm^{R} gene and a MCS. Amp^{R}, Erm^{R}	This study
P607 (pTBH07)/1464	P607 derivative, transformed with pTBH07, carrying the *ccfp* gene expressed under the constitutive promoter Phyper. Amp^{R}, Erm^{R}	This study
P610 (pTBH01)/1465	P610 derivative, transformed with pTBH01 a pACYC177 derivative containing an Erm^{R} gene and a MCS. Amp^{R}, Erm^{R}	This study
P610 (pTBH07)/1466	P610 derivative, transformed with pTBH07, carrying the *ccfp* gene expressed under the constitutive promoter Phyper. Amp^{R}, Erm^{R}	This study
P621 (pTBH01)/1467	P621 derivative, transformed with pTBH01 a pACYC177 derivative containing an Erm^{R} gene and a MCS. Amp^{R}, Erm^{R}	This study
P621 (pTBH07)/1468	P621 derivative, transformed with pTBH07, carrying the *ccfp* gene expressed under the constitutive promoter Phyper. Amp^{R}, Erm^{R}	This study
P656 (pTBH01)/1469	P597 derivative, transformed with pTBH01 a pACYC177 derivative containing an Erm^{R} gene and a MCS. Amp^{R}, Erm^{R}	This study
P656 (pTBH07)/1470	P597 derivative, transformed with pTBH07, carrying the *ccfp* gene expressed under the constitutive promoter Phyper. Amp^{R}, Erm^{R}	This study
86-028NP (pTBH03)/1455	86-028NP derivative transformed with pTBH03, carrying the *gfpmut2* gene expressed under the constitutive promoter Phyper. Amp^{R}, Erm^{R}	This study
Salmonella typhimurium		
S. typhimurium/ P1437	14028, ATCC strain	ATCC
S. typhimurium (pTBH03)/1438	*S. typhimurium* derivative, transformed with pTBH03, carrying the *gfpmut2* gene expressed under the constitutive promoter Phyper. Amp^{R}, Erm^{R}	This study

S. typhimurium (pTBH04)/1439	*S. typhimurium* derivative, transformed with pTBH04, carrying the *mcherry* gene expressed under the constitutive promoter Phyper. Amp^R, Erm^R	This study
S. typhimurium (pTBH05)/1440	*S. typhimurium* derivative, transformed with pTBH05, carrying the *mplum* gene expressed under the constitutive promoter Phyper. Amp^R, Erm^R	This study
S. typhimurium (pTBH06)/1441	*S. typhimurium* derivative, transformed with pTBH06, carrying the *morange* gene expressed under the constitutive promoter Phyper. Amp^R, Erm^R	This study

Table S2. List of primers used in this study.

Primer name	Primer ID	Primer sequence (5'-3')	Purpose
Erm_Fw_NarI		GCATGCAAGGTTTCCTAAAATCAGTGAAAA	pTBH01
Erm_Rv_XhoI_BglII		ACTAGTCATATGTATATCTCCTTCTTAAATCTGAATTCAACAATTAAATTACTTTATCATTTGCGT	pTBH01
Seq_primer_univ_AT_NheI		GCTAGCCGTATTACCGCCTTTGAGTG	pTBH01
pCN_univ_rv_AT		GTTTTGGTTCATCTTCTGTTAACTTACTAA	pTBH01
mCherry_Fw_SpeI	2158	ggACTAGTAAGGGCGAGGAGGATAACATG	pTBH04
mCherry_Rv_AscI	2159	ggGGCGCGCCTTACTTGTACAGCTCGTCCATG	pTBH04/pTBH06
CFP_Fw_SpeI	2160	ggGGCGCGCCTTACTTGTACAGCTCGTCCATG	pTHB07
CFP_Rv_AscI	2161	ggGGCGCGCCTTATTTGTAGAGTTCATCCATGCC	pTBH07
mPlum_Fw_SpeI	2194	gggACTAGTAAGGGCGAGGAGGTCATCAAGG	pTBH05
mPlum_Rv_AscI	2195	gggGGCGCGCCTTAGGCGCCGGTGGAGTGGC	pTBH05
mOrange_Fw_SpeI	2196	ggACTAGTAAGGGCGAGGAGAATAACATG	pTBH06
Lux_Fw_EcoRI	2178	ggGAATTCAGGAGGAAAAACATATGACTAAAAAAATTTC	pTBH08
Lux_Rv_AscI	2179	ggGGCGCGCCTCAACTATCAAACGCTTCGG	pTBH08
lux_int_Rv	2198	CGCATAATTTCTTTTGCGAGTG	pTBH08 checking
LB74	2088	AAGTGTTGGCCATGGAACAG	PCR checking and sequencing
pTBH-seq-fw	2089	CTGACACCCTCATCAGTGC	PCR checking and sequencing
pTBH-seq_rv	2170	AGGCATTAGTGCATTTAGACGT	PCR checking and sequencing

Table S3. Antibiotic minimal inhibitory concentration (MIC).

	**MIC (µg/mL)**	
**Antibiotic**	**NTHi RdKW20**	**NTHi R2866**
Ampicillin	0.25	16
Cefuroxime	1	1
Cefepime	≤0.25	≤0.25
Cefotaxime	≤0.06	≤0.06
Ceftriaxone	≤0.12	≤0.12
Imipenem	0.5	0.25
Meropenem	≤0.25	≤0.25
Chloramphenicol	≤0.1	≤0.1
Tetracycline	≤1	≤1
Azithromycin	4	2
Rifampicin	0.5	<1
Nalidixic acid	≤4	≤4
Levofloxacin	≤0.5	≤0.5

	**MIC (µg/mL)**	
**Antibiotic**	**NTHi RdKW20-pTBH**	**NTHi R2866-pTBH**
Ampicillin	32	>256

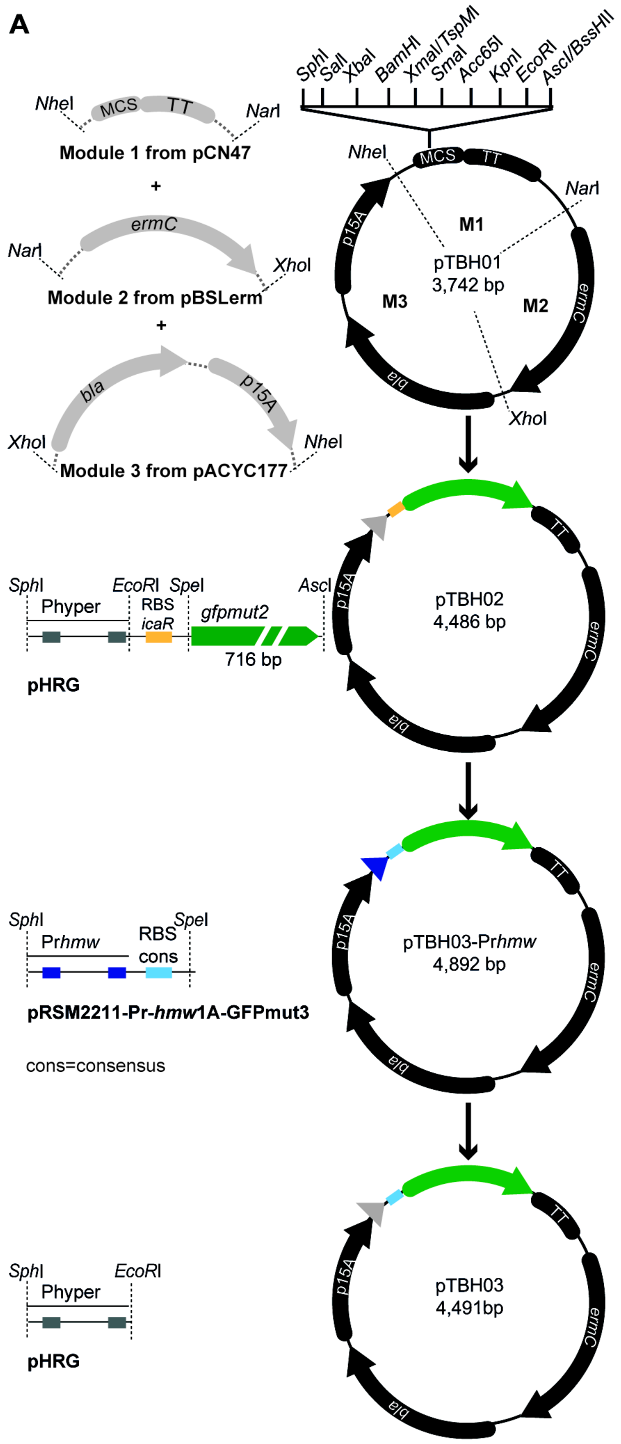

Figure S1. Schematic representation of pTBH plasmid architecture. Left panels represent the modules used for plasmid assembly. Modules (M) 1 to 3 (grey) conformed pTBH01. Next, pTBH02 was generated by cloning a *Sph*I-*Asc*I fragment excised from pHRG containing the Phyper constitutive promoter (grey portion), a ribosomal binding site (RBS) from Gram-positive bacteria (yellow portion), and the *gfpmut2* gene (green portion). pTBH03-Pr*hmw* was generated by *Sph*I-*Spe*I cloning the Pr*hmw* promoter (dark blue) and an RBS from Gram-negative bacteria (light blue) into pTBH02. Finally, Pr*hmw* was exchanged by Phyper (*Sph*I and *EcoR*I flanking sites) to generate pTBH03. pTBH01, pTBH02 and pTBH03-Pr*hmw* were built in (6), and used here to systematic generation of the pTBH series.

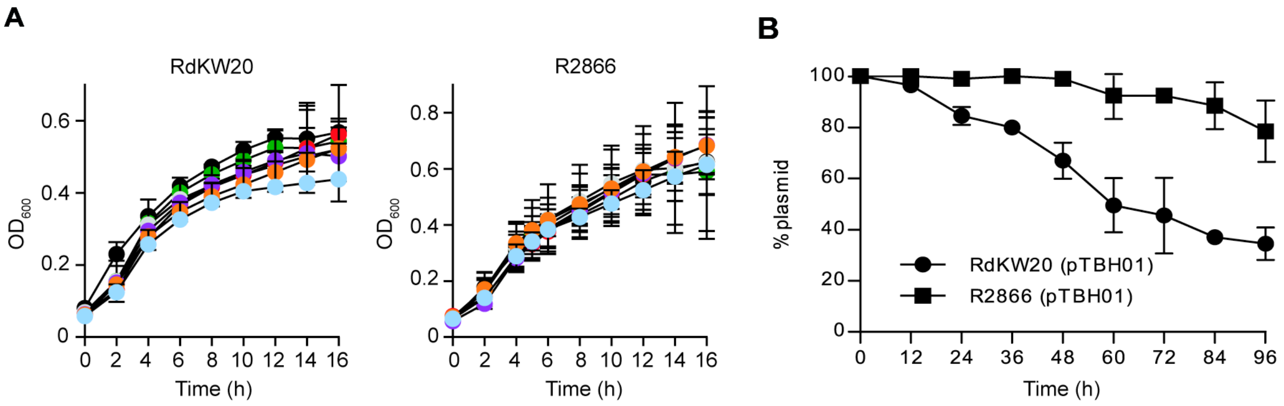

Figure S2. *H. influenzae* planktonic growth and plasmid stability assays. (A) Planktonic growth. RdKW20 (left) and R2866 (right) strains untransformed (black circle) or individually transformed with plasmids pTBH01 (grey circle), pTHB03 to 07 (color code corresponds to each respective fluorescent protein), were grown in sBHI, containing Erm$_{11}$ when needed. Strains were grown in 96-well plates, OD$_{600}$ was recorded every 30 min for 16 h; for simplicity, OD$_{600}$ values measured every 2 h are shown. RdKW20-pTBH07 growth was lower than the untransformed RdKW20 strain (from 2 to 16 h, $p<0,05$). Data are represented as mean ± SD. Statistical comparisons of means were performed by two-way ANOVA and Tukey´s multiple comparison test. **(B)** Stability of the pTBH01 plasmid. Cultures of *H. influenzae* RdKW20-pTBH01 and R2866-pTBH01 were propagated every 12 h for eight serial passages in medium without antibiotics. Eighty (R2866-pTBH01) or forty (RdKW20-pTBH01) percent of the colonies remained resistant to Erm$_{11}$ after the eighth subcultures and maintained pTBH01, confirmed by plasmid extraction and PCR mapping.

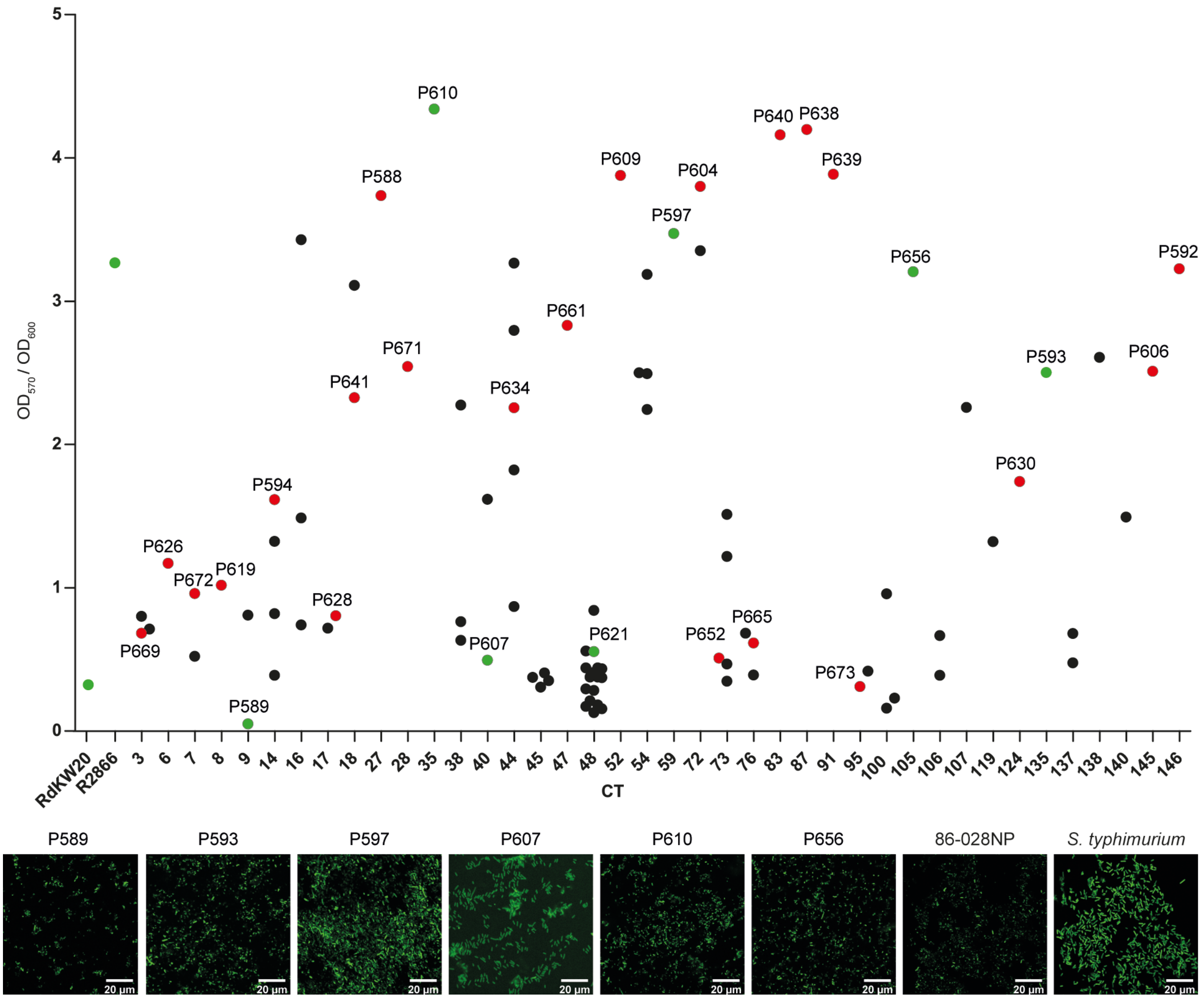

Figure S3. *H. influenzae* biofilm growth on abiotic surfaces. (A) Biofilm growth by a collection of *H. influenzae* clinical isolates. Reference strain RdKW20 and the R2866 otitis media clinical isolate (5) are included. Strains are distributed by previously established clonal types (CT) (7). Each strain is represented by one circle. Strains labelled in green or in red were used for pTBH electroporation. Successfully transformed strains are shown in green; in those cases where transformants were not obtained, strains are shown in red. The complete list of pTBH transformed derivative strains is shown in **Table S1**. **(B)** Exponentially grown fluorescent bacteria were observed by CLSM. Representative images of strains transformed with pTBH03 plasmid are shown, corresponding to six NTHi COPD clinical isolate and the 86-28NP otitis media strain (1). To expand the potential of these plasmids, a different Gram-negative bacterial species, *Salmonella typhimurium* 14028 was transformed with pTBH03; a representative image is shown on the right panel.

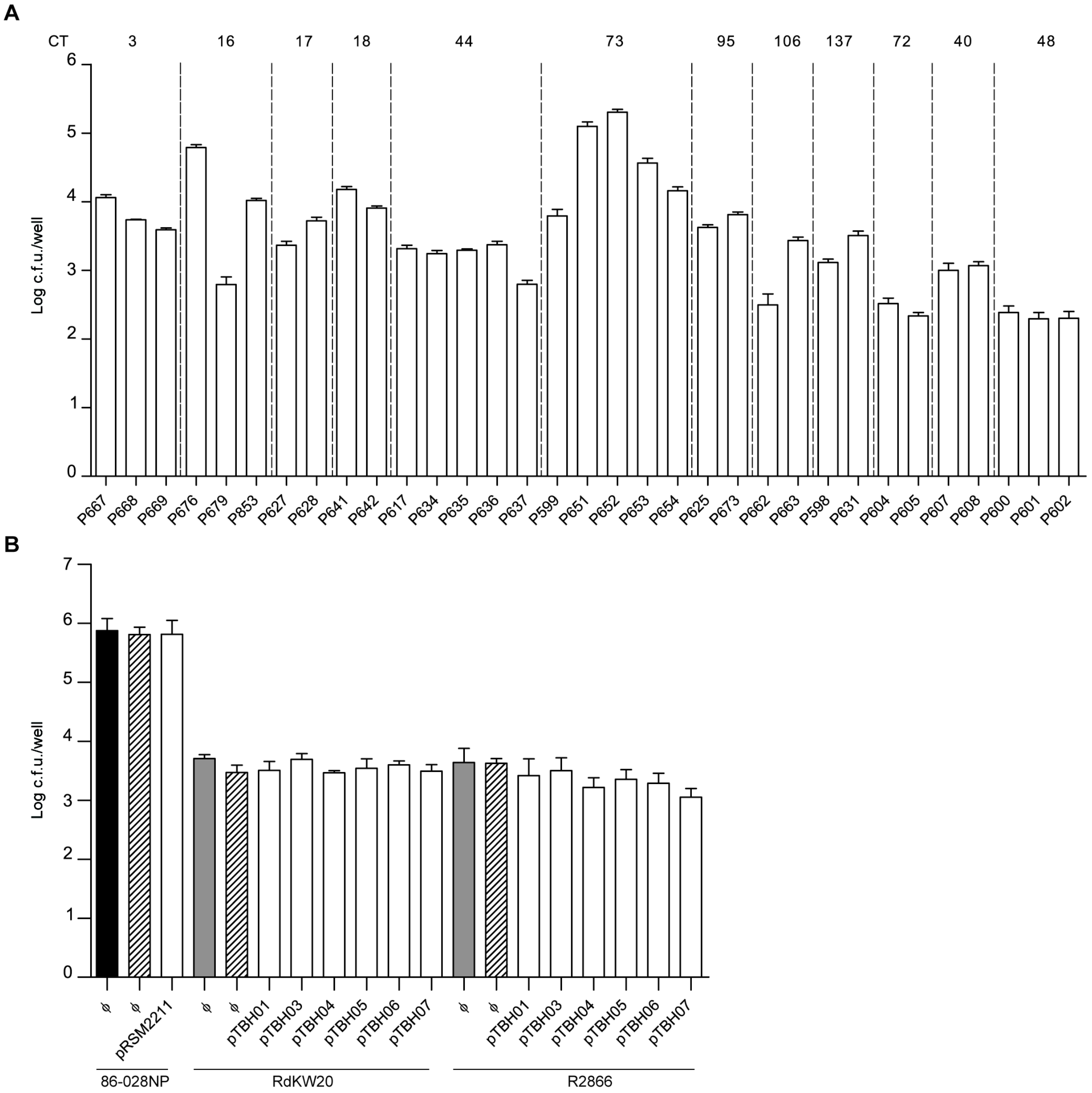

Figure S4. Invasion of A549 type II pneumocytes by *H. influenzae* strains. (A) Screening of cell invasion by a collection of *H. influenzae* clinical isolates. Strains are distributed in previously established clonal types (CT) (7). Infection was carried out with stationary phase grown bacteria. **(B)** Cell invasion by *H. influenzae* 86-028NP, in the absence (Ø) or presence (pRSM2211-GFP) of fluorescent labelling; RdWK20, in the absence (Ø, pTBH01) or presence (pTBH03-07) of fluorescent labelling; R2866, in the absence (Ø, pTBH01) or presence (pTBH03-07) of fluorescent labelling. Normalized infecting bacteria were previously grown to stationary (black, grey and white bars) or exponential (dashed bars) phase. Results are shown as Log c.f.u./well (mean±SEM). Statistical comparisons of means were performed by one-way ANOVA and Bartlett's test for equal variances. No significant differences were found.

References

1. Harrison A, Dyer DW, Gillaspy A, Ray WC, Mungur R, Carson MB, Zhong H, Gipson J, Gipson M, Johnson LS, Lewis L, Bakaletz LO, Munson Jr. RS. 2005. Genomic sequence of an otitis media isolate of nontypeable *Haemophilus influenzae*: comparative study with *H. influenzae* serotype d, strain KW20. J Bacteriol 187:4627–4636.
2. Mason KM, Munson Jr. RS, Bakaletz LO. 2003. Nontypeable *Haemophilus influenzae* gene expression induced *in vivo* in a chinchilla model of otitis media. Infect Immun 71:3454–3462.
3. Fleischmann RD, Adams MD, White O, Clayton RA, Kirkness EF, Kerlavage AR, Bult CJ, Tomb JF, Dougherty BA, Merrick JM, McKenney K, Sutton G, FitzHugh W, Fields C, Gocayne JD, Scott J, Shirley R, Liu LI, Glodek A, Kelley JM, Weidman JF, Phillips CA, Spriggs T, Hedblom E, Cotton MD, Utterback TR, Hanna MC, Nguyen DT, Saudek DM, Brandon RC, Fine LD, Fritchman JL, Fuhrmann JL, Geoghagen NSM, Gnehm CL, McDonald LA, Small K V., Fraser CM, Smith HO, Venter JC. 1995. Whole-genome random sequencing and assembly of *Haemophilus influenzae* Rd. Science (80-) 269:496–512.
4. Rodríguez-Arce I, Morales X, Ariz M, Euba B, López-López N, Esparza M, Hood DW, Leiva J, Ortíz-de-Solórzano C, Garmendia J. 2021. Development and multimodal characterization of an elastase-induced emphysema mouse disease model for the COPD frequent bacterial exacerbator phenotype. Virulence 12:1672–1688.
5. Reilly TJ, Chance DL, Smith AL. 1999. Outer membrane lipoprotein e (P4) of *Haemophilus influenzae* is a novel phosphomonoesterase. J Bacteriol 181:6797–6805.
6. Fernández-Calvet A, Euba B, Gil-Campillo C, Catalan-Moreno A, Moleres J, Martí S, Merlos A, Langereis JD, García-Del Portillo F, Bakaletz LO, Ehrlich GD, Porsch EA, Menéndez M, Mell JC, Toledo-Arana A, Garmendia J. 2021. Phase variation in HMW1A controls a phenotypic switch in *Haemophilus influenzae* associated with pathoadaptation during persistent infection. MBio e0078921.
7. Moleres J, Fernández-Calvet A, Ehrlich RL, Martí S, Pérez-Regidor L, Euba B, Rodríguez-Arce I, Balashov S, Cuevas E, Liñares J, Ardanuy C, Martín-Santamaría S, Ehrlich GD, Mell JC, Garmendia J. 2018. Antagonistic pleiotropy in the bifunctional surface protein FadL (OmpP1) during adaptation of *Haemophilus influenzae* to chronic lung infection associated with chronic obstructive pulmonary disease. MBio 9:1–23.
